# Systemic Candesartan Treatment Modulates Behavior, Synaptic Protein Levels, and Neuroinflammation in Female Mice That Express Human *APOE4*

**DOI:** 10.3389/fnins.2021.628403

**Published:** 2021-02-10

**Authors:** Sarah B. Scheinman, Steve Zaldua, Adedoyin Dada, Kateryna Krochmaliuk, Katherine Dye, Felecia M. Marottoli, Gregory R. J. Thatcher, Leon M. Tai

**Affiliations:** ^1^Department of Anatomy and Cell Biology, University of Illinois at Chicago, Chicago, IL, United States; ^2^UICentre, University of Illinois at Chicago, Chicago, IL, United States; ^3^Department of Pharmacology and Toxicology, College of Pharmacy, University of Arizona, Tucson, AZ, United States

**Keywords:** angiotensin receptor blocker, *ApoE4*, female sex, memory, inflammation

## Abstract

Evidence suggests that angiotensin receptor blockers (ARBs) could be beneficial for Alzheimer’s disease (AD) patients independent of any effects on hypertension. However, studies in rodent models directly testing the activity of ARB treatment on behavior and AD-relevent pathology including neuroinflammation, Aβ levels, and cerebrovascular function, have produced mixed results. *APOE4* is a major genetic risk factor for AD and has been linked to many of the same functions as those purported to be modulated by ARB treatment. Therefore, evaluating the effects of ARB treatment on behavior and AD-relevant pathology in mice that express human *APOE4* could provide important information on whether to further develop ARBs for AD therapy. In this study, we treated female and male mice that express the human *APOE4* gene in the absence (E4FAD−) or presence (E4FAD+) of high Aβ levels with the ARB prodrug candesartan cilexetil for a duration of 4 months. Compared to vehicle, candesartan treatment resulted in greater memory-relevant behavior and higher hippocampal presynaptic protein levels in female, but not male, E4FAD− and E4FAD+ mice. The beneficial effects of candesartan in female E4FAD− and E4FAD+ mice occurred in tandem with lower GFAP and Iba1 levels in the hippocampus, whereas there were no effects on markers of cerebrovascular function and Aβ levels. Collectively, these data imply that the effects of ARBs on AD-relevant pathology may be modulated in part by the interaction between *APOE* genotype and biological sex. Thus, the further development of ARBs could provide therapeutic options for targeting neuroinflammation in female *APOE4* carriers.

## Introduction

Alzheimer’s disease (AD) is a progressive neurodegenerative disorder and is the leading cause of dementia worldwide. Existing treatments for AD are palliative and it is therefore important to identify new therapeutic targets. This issue can be addressed by evaluating the extent that pathways modulated by known AD risk factors contribute to AD etiology regardless of risk factor status. One such risk factor is mid-life hypertension, which is often treated with angiotensin receptor blockers (ARBs). ARBs are antagonists of the Angiotensin II Type 1 (AT1) receptor and therefore limit the vasoconstrictor activity of angiotensin II (Tzourio, 2007; Kurtz and Klein, 2009). In general, evidence suggests that managing mid-life hypertension with ARBs is beneficial for AD. For example, large-scale analyses of ARB treatment in hypertensive AD patients have shown a reduction in the rate of progression of the disease (Li et al., 2010; Davies et al., 2011). Through these and other studies a key concept has emerged: that ARB treatment could be beneficial for AD patients independent of hypertension. For example, AT1 receptor signaling in the brain has been linked to AD-relevant behavioral and pathological changes *in vivo* and *in vitro* (Benicky et al., 2009; Saavedra, 2012a,b, 2016). However, studies in which AD-relevant rodent models have been directly treated with ARBs have produced mixed results at modifying memory-relevant behavioral deficits, neuroinflammation, Aβ levels, and cerebrovascular function (which we term AD-relevant pathology) (Wang et al., 2007; Mogi et al., 2008; Takeda et al., 2009; Tsukuda et al., 2009; Danielyan et al., 2010; Ferrington et al., 2011, 2012; Ongali et al., 2014, 2016; Torika et al., 2016, 2017, 2018; Royea et al., 2017; Trigiani et al., 2018). As repurposing ARBs could represent an attractive new therapy for AD, it is important to fully evaluate their activity in AD-relevant models.

Evaluating the therapeutic potential of ARBs *in vivo* can be facilitated by utilizing models that incorporate AD genetic risk factors known to modulate functions linked to AT1 receptor signaling in the brain. *APOE* genotype is the greatest genetic risk factor for the development of AD, with *APOE4* increasing AD risk up to 12-fold compared to *APOE3* (Bu, 2009; Kim et al., 2009; Holtzman et al., 2012; Huang and Mahley, 2014; Zhao et al., 2018). *APOE* can modulate neuronal function in the brain through a number of direct or indirect pathways both in the absence (Wolf et al., 2013) and presence (Carter, 2005; Ye et al., 2005; Tai et al., 2015) of Aβ. *In vivo* models have identified that *APOE4* is associated with memory-relevant behavioral deficits (Bour et al., 2008; Liu et al., 2015), greater neuroinflammation (Guo et al., 2004), higher Aβ levels (LaDu et al., 1994; Ye et al., 2005; Youmans et al., 2012; Tai et al., 2013, 2014b; Lewandowski et al., 2020), and more severe cerebrovascular dysfunction (Thomas et al., 2016, 2017; Zaldua et al., 2020), compared to *APOE3*. As described above, these are the same functions and pathologies purported to be altered by ARB treatment in mouse models expressing familial AD (FAD) mutations. Therefore, evaluating the effects of ARB treatment on AD-relevant pathology in mice that express human *APOE4* could provide important information on whether to further develop ARBs for AD therapy.

The goal of this study was to evaluate the activity of longer-term ARB treatment at modulating behavior and other pathological changes that are relevant for AD in mice that express human *APOE4*. To this end, we treated female and male mice that express the human *APOE4* gene in the absence (E4FAD−) or presence (E4FAD+) of Aβ overproduction (Youmans et al., 2012; Tai et al., 2017) with the ARB prodrug candesartan cilexetil for a duration of 4 months and assessed the following: plasma drug levels, blood pressure, memory-relevant behavior, synaptic protein levels, angiotensin peptides and receptors, neuroinflammation, Aβ levels, and markers of cerebrovascular function.

## Materials and Methods

### Mouse Model and Treatment

All experiments follow the UIC Institutional Animal Care and Use Committee protocols. E4FAD mice were produced by crossing mice that express 5 Familial Alzheimer’s Disease (5xFAD) mutations (APP K670N/M671L + I716 V + V717I and PS1 M146L + L286 V) with *APOE4*-targeted replacement mice (Youmans et al., 2012). *APOE4*-targeted replacement mice are homozygous for the human *APOE4* gene. E4FAD carrier mice are *APOE*^+/+^/5xFAD^+/–^ (E4FAD+) and non-carrier mice are *APOE*^+/+^ 5xFAD^–/–^ (E4FAD−). Female and male E4FAD− and E4FAD+ mice were used in this study as identified by genotyping of tail samples (see Supplementary Figure 1 for full *n* sizes).

Treatments were administered in hydrogel which replaces drinking water as described in Tai et al. (2014a). Each mouse (∼30 *g*) consumes roughly 4.5 ml of hydrogel per day; consumption was routinely monitored, and hydrogel was replaced three times a week to ensure fresh drug was consistently available. Candesartan cilexetil (Cayman Chemical Company) was dissolved in molecular biology grade DMSO and mixed in hydrogel (Clear H_2_O Inc.). Mice received hydrogel containing vehicle (0.22% DMSO), 0.0067 mg/ml (1 mg/kg/day) candesartan cilexetil, or 0.067 mg/ml (10 mg/kg/day) candesartan cilexetil. Female mice were treated from 6 months of age until 10 months of age, and male mice were treated from 8 months of age until 12 months of age. All behavioral testing was conducted blinded, however, due to issues related to COVID19, biochemical and IHC analysis was conducted unblinded.

### Behavioral Testing

Behavioral analysis was conducted in the mouse dark cycle, tracked in real time by a camera, and analyzed using Any-Maze software as described in Thomas et al. (2016, 2017), Marottoli et al. (2017, 2019).

#### Open Field

A single mouse was placed in the center of a white acrylic container (l 432 mm × w 305 mm× h 300 mm) covered with bedding and allowed to freely explore for 7 min. Total distance traveled and percent distance traveled in perimeter of the container were calculated (Thomas et al., 2016, 2017; Marottoli et al., 2017, 2019).

#### Novel Object Recognition

Open field was conducted 1 day prior to novel object recognition and therefore served as the habitation phase. Mice were placed in the center of the same testing chamber as described for the open field test, containing two identical objects placed equidistant from each other and the walls of the box, and allowed to explore for 7 min. Mice were then returned to their home cage for 1 h, after which they were placed in the testing chamber for an additional 7 min with a familiar and a novel object. Total investigation time of both objects as well as the preference index (ratio of time spent with the novel object divided by the total investigation time of both objects) were calculated for both phases of the test (Thomas et al., 2016, 2017; Marottoli et al., 2017, 2019). Mice that had a total investigation time of less than 20 s were excluded from analysis.

### Blood Pressure Measurement

Blood pressure was measured using a noninvasive tail-cuff system (Kent Scientific Company). Mean arterial pressure was assessed using Volume Pressure Recording (VPR) sensor technology. Mice were habituated to the restraining device and tail cuffs for 20 min a day for 2 days prior to the testing day. Five additional acclimation cycles were performed before acquiring 15 measurements of mean arterial pressure which were then averaged to determine each animal’s blood pressure.

### Tissue Processing

Mice were deeply anesthetized with 150 μl ketamine and 50 μl xylazine (i.p), blood was drawn by cardiac puncture, and transcardial perfusion was performed using ice-cold PBS. Dissected left hemi-brains were frozen in O.C.T and stored at −80°C until processing for immunohistochemical (IHC) analysis. Right hemi-brains were further dissected into the hippocampus, which was then flash frozen in liquid nitrogen and stored at −80°C until processing for biochemical analysis.

### Biochemical Analysis

Hippocampal samples were weighed and extracted using a 2-step extraction protocol with modifications as described previously (Youmans et al., 2012; Tai et al., 2013, 2014a; Thomas et al., 2016, 2017; Marottoli et al., 2017, 2019), in order to separate out soluble and detergent-soluble proteins. Briefly, samples were homogenized using a bead mill (Fisherbrand) at 6 m/s for 1 cycle of 30 s in ice cold TBS at 10 μl/mg of brain tissue, centrifuged (100,000 × *g* for 30 min), and aliquoted. The resulting pellet was then resuspended in SDS buffer (1% SDS + 10 mM NaF + 2 mM Na_3_VO_4_ + 1× protease inhibitor cocktail in 20 mM HEPES; pH = 7.4), mixed via end-over-end rotation for 30 min at 4°C, sonicated (20% amplification, 3 cycles), centrifuged (100,000 × *g* for 30 min), and aliquoted. TBS and SDS buffer aliquots were flash frozen in liquid nitrogen and stored at −80°C. Total protein was quantified in TBS and SDS buffer extracts using the Pierce BCA Protein Assay Kit.

#### Western Blot Analysis

SDS buffer fractions were analyzed using western blot as described previously (Thomas et al., 2016, 2017; Marottoli et al., 2017, 2019). Briefly, twenty micrograms of protein were separated on 4–12% Bis-Tris gels (Invitrogen), transferred onto low-fluorescence PVDF membranes, blocked with 5% milk in TBS for 1 h at room temperature, washed with 0.1% Tween-20 in TBS (TBS-T), and probed with primary antibodies (see Supplementary Table 1) in 1% bovine serum albumin in TBS with 0.02% Sodium Azide overnight at 4°C. After washing (3 × 5 min, TBS-T), membranes were incubated for 45 min in appropriate secondary florescent antibodies (see Supplementary Table 1) in 1% milk in TBST and 0.01% SDS. All proteins were imaged and quantified using the Odyssey Fc Imaging System and normalized to GAPDH.

#### ELISA Analysis

Apolipoprotein E (ApoE), Aβ42, and Angiotensin II levels were measured in hippocampal homogenates by ELISA. The apoE ELISA was performed using anti-apoE (1:2000, Millipore) and biotinylated anti-apoE (1:5000, Meridian) for capture and detection antibodies as described in Thomas et al. (2016, 2017), Marottoli et al. (2017). Aβ42 (Life Technologies) and Angiotensin II (Cloud-Clone Corp.) were measured following the manufacturer instructions. ApoE, Aβ42, and Angiotensin II levels were normalized to total protein levels in each of the distinct fractions. In addition, we calculated total Aβ42 and total apoE levels using the following equation:

(1) $$\frac{\left(T\,o\,t\,a\,l\,A\,\beta \,42\,T\,B\,S+T\,o\,t\,a\,l\,A\,\beta \,42\,S\,D\,S\,b\,u\,f\,f\,e\,r\right)}{\left(T\,o\,t\,a\,l\,P\,r\,o\,t\,e\,i\,n\,T\,B\,S+T\,o\,t\,a\,l\,P\,r\,o\,t\,e\,i\,n\,S\,D\,S\,b\,u\,f\,f\,e\,r\right)}$$

#### Cytokine/Chemokine Analysis

Levels of 31 chemokine/cytokines were quantified in hippocampal homogenates (diluted in Assay Buffer to 0.1% SDS) using a Mouse cytokine/chemokine magnetic bead panel (MILLIPLEX), following the manufacturer instructions. Concentrations of individual cytokines and chemokines were calculated using Belysa software with a five-parameter logistic curve fitting method, and normalized to total protein levels.

### Immunohistochemical Analysis

Fluorescent IHC analysis was conducted as described previously (Thomas et al., 2016, 2017; Marottoli et al., 2017, 2019). Frozen brains were sectioned at 12 μm and nine nonadjacent sagittal sections (∼108 μm apart) were utilized for quantification per animal. Slides were fixed using 10% Neutral Buffered Formalin (Sigma) for 10 min, washed with PBS (3 × 5 min), incubated in 52.8% formic acid (8 min), permeabilized for 3 × 5 min with PBS containing 0.25% Triton-X (PBS-X) and blocked with 5% BSA in PBS-X for 2 h at room temperature. Slides were then incubated in primary antibody (see Supplementary Table 1) for 48 h in a humidified chamber at 4°C in PBS containing 2% BSA and 0.1% Triton-X. Slides were then washed (3 × 5 min PBS-X), incubated for 2 h at room temperature with fluorophore-conjugated secondary antibodies in PBS containing 2% BSA and 0.1% Triton-X, washed in PBS-X (3 × 5 min) and PBS (1 × 5 min), and then cover-slipped using Fluoromount-G (SouthernBiotech). Mosaic images were captured and stitched on a Molecular Devices ImageXpress Micro 4 instrument under identical capture settings at 10× magnification. Quantification was performed using MetaXpress software: images were thresholded to diminish background signal and percent area covered by each stain was calculated in the hippocampus.

### Pharmacokinetic Analysis

The concentration of candesartan in plasma was evaluated. Standard curves were established in corresponding biological matrix using olmesartan as an internal standard. The standard curve range was 1–1,000 ng/mL. Plasma samples were added to a microcentrifuge tube along with cold acetonitrile containing internal standard. The samples were vortexed, centrifuged and the supernatant was transferred to new tubes for evaporation under nitrogen gas. Once dried down, the samples were reconstituted, vortexed, centrifuged and transferred to LC vial for analysis. Candesartan and olmesartan were monitored using a Shimadzu Ultra-Fast Liquid Chromatograph with Shimadzu 8040 Triple Quadrupole Mass Spectrometer (UFLC-MS/MS). The separation was achieved by using 10% methanol in water with 0.1% formic acid and acetonitrile with 0.1% formic acid as the mobile phases and a Phenomenex Kinetex C18 column (50 × 3 mm, 2.6 μm particles) with the appropriate guard column. The triple quadrupole source was positive electrospray ionization using multiple reaction monitoring to monitor both candesartan and olmesartan. The quantitation analysis was performed using Shimadzu’s LabSolutions software, with weighting of the calibration curve model being 1/x^2^.

### Statistical Analysis

All data are presented as the mean +/− S.E.M and were analyzed using one-way ANOVA followed by Dunnett’s multiple comparisons testing, or by using Student’s *t*-test with GraphPad Prism version 8.3.1. See Supplementary Table 2 for details on *n* sizes and statistical comparisons.

## Results

The goal of this study was to evaluate the activity of longer-term candesartan cilexetil treatment at modulating behavior and other markers of AD-relevant pathology in mice that express human *APOE4*. To address this goal, female (6–10-month treatment duration) and male (8–12-month treatment duration) E4FAD− and E4FAD+ mice were treated with either vehicle (0 mg/kg/day), 1 mg/kg/day candesartan cilexetil, or 10 mg/kg/day candesartan cilexetil in hydrogel. The impact of candesartan cilexetil (referred to as candesartan) treatment on the following was then assessed: plasma drug levels, blood pressure, memory-relevant behavior, synaptic protein levels, angiotensin peptides and receptors, neuroinflammation, Aβ levels, and cerebrovascular dysfunction (full study design outlined in Supplementary Figure 1).

E4FAD− and E4FAD+ mice were selected for this study as they express human *APOE4* and exhibit age-related changes in memory-relevant behavior and functions described as potential targets of ARBs (AD-relevant pathology: neuroinflammation, Aβ levels, and cerebrovascular dysfunction; Youmans et al., 2012; Tai et al., 2017). E4FAD− mice express the human *APOE4* gene under the endogenous mouse promoter, and E4FAD+ mice express the human *APOE4* gene and overproduce human Aβ42 through the expression of 5xFAD autosomal dominant mutations. *APOE4* is associated with age-dependent changes in behavior and neuronal function in the absence of high levels of human Aβ *in vivo*, including in E4FAD− mice (Wolf et al., 2013; Thomas et al., 2016, 2017; Zaldua et al., 2020). Therefore, E4FAD− and E4FAD+ mice were utilized to test whether candesartan modulates functions specific to the interaction of Aβ and *APOE4* or were generally applicable to *APOE4*. Previous research in E4FAD− and E4FAD+ mice has demonstrated that alterations in behavior and neuronal protein levels occurs earlier in female mice (∼6 months) than in male mice (∼8 months) (Wolf et al., 2013; Thomas et al., 2016, 2017; Tai et al., 2017; Zaldua et al., 2020). Therefore, candesartan treatments were initiated at different ages for female and male E4FAD mice to try and broadly match AD-relevant pathology at the start of treatment. As our pilot data demonstrated that a 100 mg/kg/day dose of candesartan produced overt signs of toxicity in mice, a 10 mg/kg/day dose and a 1 mg/kg/day dose were selected to assess differences in treatment with a high versus a low dose of the drug. Based on our primary question, statistical analysis was conducted using within group comparisons, i.e., comparing vehicle treatment to candesartan treatment within each genotype for a particular sex (see Supplementary Table 2 for details of statistical analysis).

### High-Dose Candesartan Treatment Resulted in Higher Plasma Candesartan Levels and Lower Blood Pressure Compared to Vehicle Treatment in E4FAD− and E4FAD+ Mice

**FIGURE 1:**
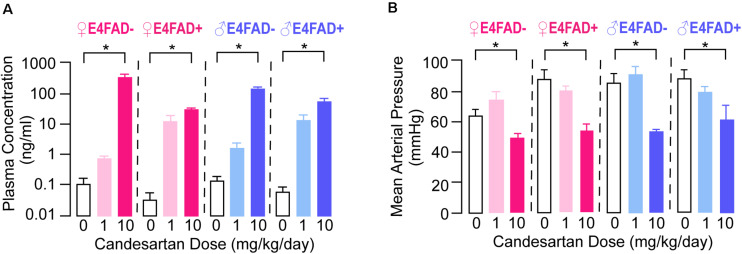
Female and male E4FAD− and E4FAD+ mice treated with high-dose candesartan had higher plasma levels of candesartan and lower blood pressure compared to vehicle. **(A)** Female and male E4FAD− and E4FAD+ mice treated with 10 mg/kg/day candesartan (high dose) had higher plasma drug levels than mice treated with vehicle (0 mg/kg/day) [Female E4FAD−: *F*(2, 6) = 13.38, *p* = 0.006. Female E4FAD+: *F*(2, 5) = 19.10, *p* = 0.0046. Male E4FAD−: *F*(2, 4) = 324.6, *p* < 0.0001. Male E4FAD+: *F*(2, 6) = 13.53, *p* = 0.006]. **(B)** Female and male E4FAD− and E4FAD+ mice treated with 10 mg/kg/day candesartan had lower mean arterial blood pressure at treatment endpoint compared to vehicle (Female E4FAD−: *F*(2, 19) = 6.54, *p* = 0.0069. Female E4FAD+: *F*(2, 22) = 11.95, *p* = 0.0003. Male E4FAD−: *F*(2, 20) = 14.04, *p* = 0.0002. Male E4FAD+: *F*(2, 24) = 4.56, *p* = 0.021). All data expressed as mean +/− SEM. *p < 0.05 by one-way ANOVA and Dunnet’s *post-hoc* analysis for candesartan dose compared to vehicle. See Supplementary Table 2 for details on *n* size and statistical comparisons.

We initially evaluated the extent that candesartan entered the systemic circulation using plasma pharmacokinetic analysis. Candesartan as the prodrug was administered continuously *ad libitum* in hydrogel, mimicking an extended-release formulation. In all experimental groups (both female and male, E4FAD−, and E4FAD+ mice), plasma drug levels were greater than 10 ng/ml in mice treated with 10 mg/kg/day candesartan (referred to as high-dose; Figure 1A). In mice treated with 1 mg/kg/day candesartan (referred to as low-dose), drug levels in plasma were variable and in many samples were not above the limit of detection (LOD) of the LC-MS/MS method used. The variance in plasma drug levels in the low-dose candesartan treatment condition may reflect the *ad libitum* mode of drug-in-hydrogel administration, with some mice consuming hydrogel immediately prior to sacrifice while others did not, which is consistent with the significant, but relatively low concentration measured in the high dose group. The prodrug itself, candesartan cilexetil, was not observed in any samples above its LOD.

An additional method for evaluating plasma drug bioavailability is to assess a peripheral pharmacodynamic readout associated with the drug target. Candesartan was originally developed as a medication to lower blood pressure in patients with hypertension, which may also occur under normotensive conditions. Therefore, we evaluated the effects of candesartan treatment on mean arterial blood pressure in a subset of female and male E4FAD− and E4FAD+ mice at treatment endpoint (Figure 1B). High-dose, but not low-dose, candesartan treatment resulted in lower blood pressure in female and male E4FAD− and E4FAD+ mice compared to vehicle treatment. Indeed, mean arterial pressure was ∼15–32% lower with high-dose candesartan treatment as compared to vehicle treatment. These data suggest that the high-dose treatment of candesartan, resulting in measurable plasma drug levels, induced hypotension in female and male E4FAD− and E4FAD+ mice.

Taken together, our pharmacokinetic and pharmacodynamic data demonstrate that high-dose candesartan treatment, using our treatment regimen, resulted in plasma concentrations in female and male E4FAD− and E4FAD+ mice sufficient to elicit an on-target pharmacodynamic effect.

### High-Dose Candesartan Treatment Was Beneficial for Memory-Relevant Behavior and Resulted in Higher Hippocampal Synaptic Protein Levels in Female E4FAD− and E4FAD+ Mice

**FIGURE 2:**
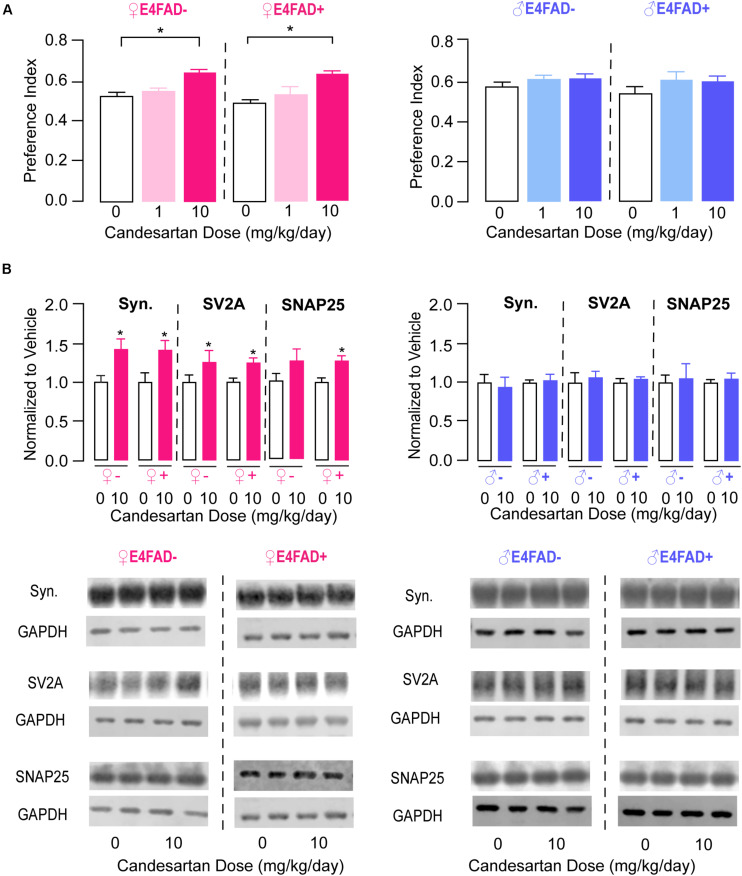
High-dose candesartan treatment resulted in improved memory-relevant behavior and higher hippocampal presynaptic protein levels in female, but not male, E4FAD− and E4FAD+ mice. **(A)** In female E4FAD− and E4FAD+ mice treated with 10 mg/kg/day (high dose) candesartan, performance was greater in the novel object recognition test compared to vehicle (0 mg/kg/day) at treatment endpoint [Female E4FAD−: *F*(2, 25) = 15.29, *p* < 0.0001. Female E4FAD+: *F*(2, 42) = 14.64, *p* < 0.0001]. This effect was not seen in male E4FAD− and E4FAD+ mice treated with 10 mg/kg/day candesartan [Male E4FAD−: *F*(2, 23) = 1.51, *p* = 0.24. Male E4FAD+: *F*(2, 26) = 1.23, *p* = 0.31]. **(B)** Female E4FAD− and E4FAD+ mice treated with 10 mg/kg/day candesartan had higher hippocampal levels of synaptophysin [Female E4FAD−: *t*(17) = 2.65, *p* = 0.017. Female E4FAD+: *t*(19) = 2.89, *p* = 0.0092], SV2A [Female E4FAD−: *t*(15) = 2.33, *p* = 0.034. Female E4FAD+: *t*(17) = 2.83, *p* = 0.012], and SNAP25 [Female E4FAD−: *t*(17) = 1.45, *p* = 0.16. Female E4FAD+: *t*(20) = 3.03, *p* = 0.0067] compared to vehicle when assessed by western blot analysis. Male E4FAD− and E4FAD+ mice treated with 10 mg/kg/day candesartan did not have altered hippocampal levels of synaptophysin [Male E4FAD−: *t*(10) = 0.33, *p* = 0.75. Male E4FAD+: *t*(9) = 0.34, *p* = 0.74], SV2A [Male E4FAD−: *t*(10) = 0.43, *p* = 0.67. Male E4FAD+: *t*(9) = 0.66, *p* = 0.53], or SNAP25 [Male E4FAD−: *t*(10) = 0.25, *p* = 0.81. Male E4FAD+: *t*(9) = 0.56, *p* = 0.59] compared to vehicle. Quantification of each protein was normalized to GAPDH as a loading control and all data are expressed as a ratio to vehicle-treated mice. All data expressed as mean +/− SEM. *p < 0.05 by one-way ANOVA and Dunnet’s *post-hoc* analysis for candesartan dose comparisons to vehicle **(A)** and by Student’s *t*-test **(B)**. See Supplementary Table 2 for details on *n* sizes and statistical comparisons.

Impaired memory-relevant behavior with *APOE4* has been reported both in the presence and absence of Aβ *in vivo* (Thomas et al., 2016, 2017; Zaldua et al., 2020), and data are conflicting on whether ARB treatment considerably modulates memory-relevant behavior in AD-relevant mouse models (Wang et al., 2007; Mogi et al., 2008; Takeda et al., 2009; Tsukuda et al., 2009; Danielyan et al., 2010; Ferrington et al., 2011, 2012; Ongali et al., 2014, 2016; Torika et al., 2016, 2017, 2018; Royea et al., 2017; Trigiani et al., 2018). Therefore, one of our goals was to evaluate whether candesartan treatment could modulate memory-relevant behavior in E4FAD− and E4FAD+ mice. We initially identified that candesartan treatment (low-dose and high-dose) did not alter open field performance. Compared to vehicle treatment, with high-dose candesartan treatment there were no changes in the total distance traveled or percent distance traveled in the perimeter of the open field arena in female and male E4FAD− and E4FAD+ mice (Supplementary Figure 2). These data support that candesartan does not alter locomotor activity or anxiety-like behavior in mice that express human *APOE4* using our treatment regimen. Next, we evaluated memory-relevant behavior using the novel object recognition test. In female E4FAD− and E4FAD+ mice, high-dose candesartan treatment resulted in a greater proportion of novel object investigation compared to vehicle treatment. Indeed, in both E4FAD− and E4FAD+ female mice treated with high-dose candesartan, performance (preference index) was ∼25–30% higher compared to mice treated with vehicle (Figure 2A, left). In male E4FAD− and E4FAD+ mice, high-dose candesartan treatment did not modulate performance in the novel object recognition test compared to vehicle treatment (Figure 2A, right).

We next explored whether candesartan treatment altered synaptic protein markers in the hippocampus of E4FAD− and E4FAD+ mice by western blot analysis. Based on novel object recognition data, we only compared high-dose candesartan treatment with vehicle treatment for female and male E4FAD− and E4FAD+ mice (i.e., omitted the low-dose candesartan groups). There were no changes in hippocampal levels of archetypal glutamatergic (Vesicular Glutamate Transporter Type 1) or GABAergic (Glutamate Decarboxylase 67) synaptic markers with candesartan treatment in any of the groups (Supplementary Figure 3). However, hippocampal levels of the general presynaptic protein markers synaptophysin, SV2A, and SNAP25 were ∼25–40% higher with high-dose candesartan treatment compared to vehicle treatment in female E4FAD− and E4FAD+ mice (Figure 2B, left). In male E4FAD− and E4FAD+ mice, there were no changes in hippocampal levels of synaptophysin, SV2A, or SNAP25 with candesartan treatment compared to vehicle treatment (Figure 2B, right). Additionally, there were no changes in cortical levels of Vglut1 or synaptophysin in female E4FAD+ (Supplementary Figure 6) with high-dose candesartan treatment compared to vehicle treatment. Thus, improvements in memory-relevant behavior associated with high-dose candesartan treatment were accompanied by higher hippocampal presynaptic protein levels in female E4FAD− and E4FAD+ mice.

### High-Dose Candesartan Treatment Did Not Alter Levels of Brain Angiotensin Peptides or Receptors in E4FAD− and E4FAD+ Mice

**FIGURE 3:**
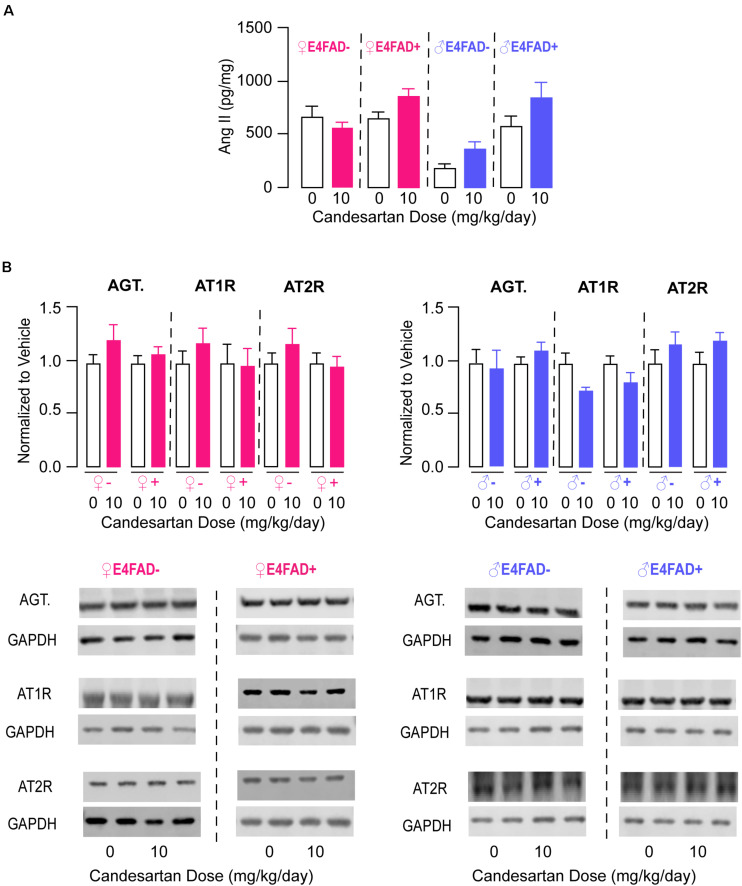
High-dose candesartan treatment did not alter hippocampal angiotensin peptide and receptor levels compared to vehicle treatment in female and male E4FAD− and E4FAD+ mice. In female and male E4FAD− and E4FAD+ mice treated with 10 mg/kg/day candesartan (high dose), there were no differences in hippocampal levels of **(A)** angiotensin II when assessed by ELISA analysis [Female E4FAD−: *t*(18) = 0.15, *p* = 0.88. Female E4FAD+: *t*(17) = 1.85, *p* > 0.081. Male E4FAD−: *t*(8) = 2.23, *p* = 0.056. Male E4FAD+: *t*(8) = 1.53, *p* = 0.16] as well as **(B)** angiotensinogen, AT1 receptor, or AT2 receptor levels compared to vehicle when assessed by western blot analysis [Angiotensinogen: Female E4FAD−: *t*(17) = 1.30, *p* = 0.21. Female E4FAD+: *t*(22) = 0.81, *p* = 0.43. Male E4FAD−: *t*(10) = 0.27, *p* = 0.79. Male E4FAD+: *t*(9) = 1.19, *p* = 0.26. AT1: Female E4FAD−: *t*(18) = 1.00, *p* = 0.33. Female E4FAD+: *t*(21) = 0.09, *p* = 0.92. Male E4FAD−: *t*(10) = 2.28, *p* = 0.073. Male E4FAD+: *t*(9) = 1.44, *p* = 0.18. AT2: Female E4FAD−: *t*(18) = 1.00, *p* = 0.33. Female E4FAD+: *t*(22) = 0.21, *p* = 0.84. Male E4FAD−: *t*(10) = 1.04, *p* = 0.32. Male E4FAD+: *t*(9) = 1.45, *p* = 0.17]. In **(B)** quantification of each protein was normalized to GAPDH as a loading control and all data are expressed as a ratio to vehicle-treated mice. All data expressed as mean +/− SEM. *p* > 0.05 by Student’s *t*-test. See Supplementary Table 2 for details on *n* sizes.

We next focused on evaluating markers of pathways that could be associated with/contribute to improvements in memory and synaptic markers with high-dose candesartan treatment in E4FAD− and E4FAD+ mice. One possibility was that high-dose candesartan altered levels of endogenous brain angiotensin peptides and receptors. Therefore, we began by measuring levels of angiotensin II by ELISA analysis. In female and male E4FAD− and E4FAD+ mice, there were no differences in hippocampal levels of angiotensin II with high-dose candesartan treatment compared to vehicle treatment (Figure 3A). Consistent with a lack of treatment effect on angiotensin II levels, there were also no differences in hippocampal levels of angiotensinogen, the peptide precursor to angiotensin II, with high-dose candesartan treatment compared to vehicle treatment in female or male E4FAD− and E4FAD+ mice when assessed by western blot analysis (Figure 3B). We next assessed whether high-dose candesartan treatment altered angiotensin II receptor levels in the hippocampus of E4FAD− and E4FAD+ mice. When assessed by western blot analysis, there were no changes in absolute levels of the AT1 receptor or AT2 receptor with high-dose candesartan treatment compared to vehicle treatment in female or male E4FAD− and E4FAD+ mice (Figure 3B). These data collectively support that the beneficial effects of high-dose candesartan treatment in E4FAD− and E4FAD+ mice occurred independent of changes in levels of angiotensin peptides or receptors in the hippocampus.

### High-Dose Candesartan Treatment Resulted in Lower Levels of Neuroinflammatory Markers in Female E4FAD− and E4FAD+ Mice

**FIGURE 4:**
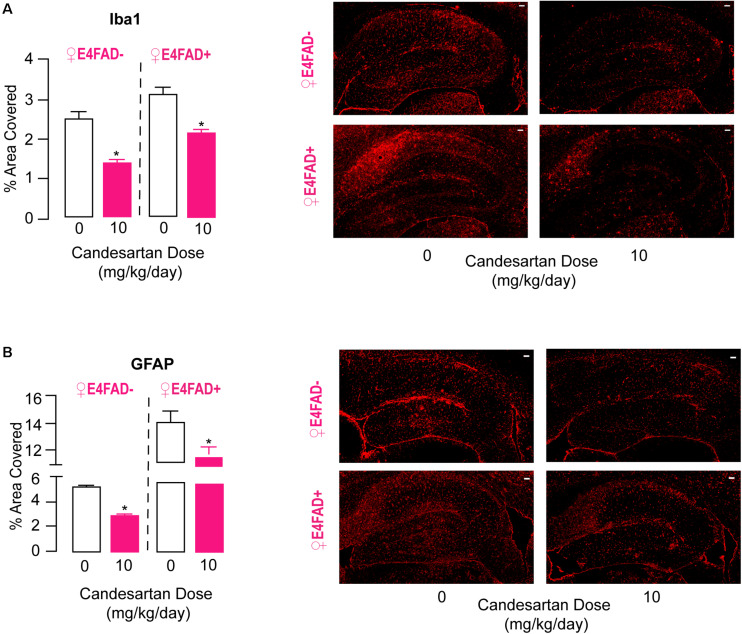
High-dose candesartan treatment resulted in lower hippocampal levels of neuroinflammatory markers compared to vehicle treatment in female E4FAD− and E4FAD+ mice. Female E4FAD− and E4FAD+ mice treated with 10 mg/kg/day (high dose) candesartan had a lower percentage of hippocampal area immunostained with **(A)** Iba1 [E4FAD−: *t*(16) = 6.76, *p* < 0.0001. E4FAD+: *t*(18) = 5.08, *p* < 0.0001] and **(B)** GFAP [E4FAD−: *t*(16) = 15.06, *p* < 0.0001. E4FAD+: *t*(18) = 2.44, *p* = 0.025] compared to vehicle (0 mg/kg/day) when assessed by quantitative IHC analysis. All data expressed as mean +/− SEM. *p < 0.05 by Student’s *t*-test. Scale bars = 100 μm. See Supplementary Table 2 for details on *n* sizes.

Activation of astrocytes and microglia in the brain, often termed gliosis, are key components of the neuroinflammatory response. One approach to evaluating gliosis is via quantification of staining for Iba1 (microglia) and GFAP (astrocytes), levels of which are both higher in AD patients (Hein and O’Banion, 2009) and ARB treatment is associated with lower GFAP and Iba1 immunoreactivity in FAD mice (Wang et al., 2007; Mogi et al., 2008; Takeda et al., 2009; Tsukuda et al., 2009; Danielyan et al., 2010; Ferrington et al., 2011, 2012; Ongali et al., 2014, 2016; Torika et al., 2016, 2017, 2018; Royea et al., 2017; Trigiani et al., 2018). Therefore, we measured hippocampal levels of Iba1 (Figure 4A) and GFAP (Figure 4B) in female E4FAD− and E4FAD+ mice by quantitative IHC analysis. Compared to vehicle treatment, with high-dose candesartan treatment there were lower levels of Iba1 (44% for E4FAD− and 30% for E4FAD+) and GFAP (43%, for E4FAD− and 16% for E4FAD+) in the hippocampus of female E4FAD− and E4FAD+ mice. However, there were no differences in cortical levels of GFAP in female E4FAD+ mice that were treated with high-dose candesartan compared to vehicle (Supplementary Figure 6B). Alterations in glial morphology is another indication of gliosis (e.g., number of processes in astrocytes, ameboid appearance for microglia); however, qualitatively we could not distinguish the morphology of GFAP-positive astrocytes between candesartan-treated and vehicle-treated female E4FAD− and E4FAD+ mice (Supplementary Figure 7). Although microglia in E4FAD+ mice appeared more ameboid than E4FAD− mice, most likely due to high Aβ levels, there were no morphological differences in hippocampal Iba1-positive microglia in female E4FAD− and E4FAD+ mice treated with candesartan compared to vehicle (Supplementary Figure 7). Thus, candesartan treatment potentially modulated the total number of activated astrocytes and microglia, rather than morphology, in the hippocampus. Since candesartan treatment improved behavior and lowered markers of gliosis, we next evaluated whether Iba1 or GFAP immunostaining correlated with memory-relevant behavior (NOR preference index) in combined data from female E4FAD− and E4FAD+ mice (Supplementary Figure 5A). In female E4FAD− and E4FAD+ female mice, there was a moderate negative correlation between hippocampal levels of Iba1, but not GFAP, and NOR performance, (Supplementary Figure 5). One way that glial activation is proposed to modulate neuron function is through the production of cytokines/chemokines; therefore, we measured the levels of 31 chemokines/cytokines in hippocampal SDS extracts of female E4FAD− and E4FAD+ mice (Supplementary Table 3). However, except for GCSF (lower in EFAD+ mice with candesartan treatment compared to vehicle) there were no changes in cytokine/chemokine levels with candesartan treatment. These data suggest that mechanistically, other glial functions are modulated by candesartan treatment to result in improved behavior and neuron function.

Overall, our data demonstrate that compared to vehicle treatment, high-dose candesartan treatment modulated hippocampal astrogliosis and microgliosis in female E4FAD− and E4FAD+ mice, which could contribute to improvements in memory-relevant behavior and synaptic protein levels.

### High-Dose Candesartan Treatment Did Not Alter Markers of Vessel Coverage or Cerebrovascular Leakiness in Female E4FAD− and E4FAD+ Mice

Cerebrovascular dysfunction is generally thought to contribute to neuronal and behavioral dysfunction during AD progression and is also one of the potential targets of ARB treatment (Wang et al., 2007; Mogi et al., 2008; Takeda et al., 2009; Tsukuda et al., 2009; Danielyan et al., 2010; Ferrington et al., 2011, 2012; Ongali et al., 2014, 2016; Torika et al., 2016, 2017, 2018; Royea et al., 2017; Trigiani et al., 2018). Previous studies have demonstrated lower vessel coverage and higher cerebrovascular leakiness in 8 month old E4FAD− (Thomas et al., 2017) and E4FAD+ mice (Tai et al., 2017) compared to mice expressing *APOE3*. Therefore, we assessed whether high-dose candesartan treatment in female E4FAD− and E4FAD+ mice modulated these markers of cerebrovascular dysfunction using quantitative IHC analysis.

**FIGURE 5:**
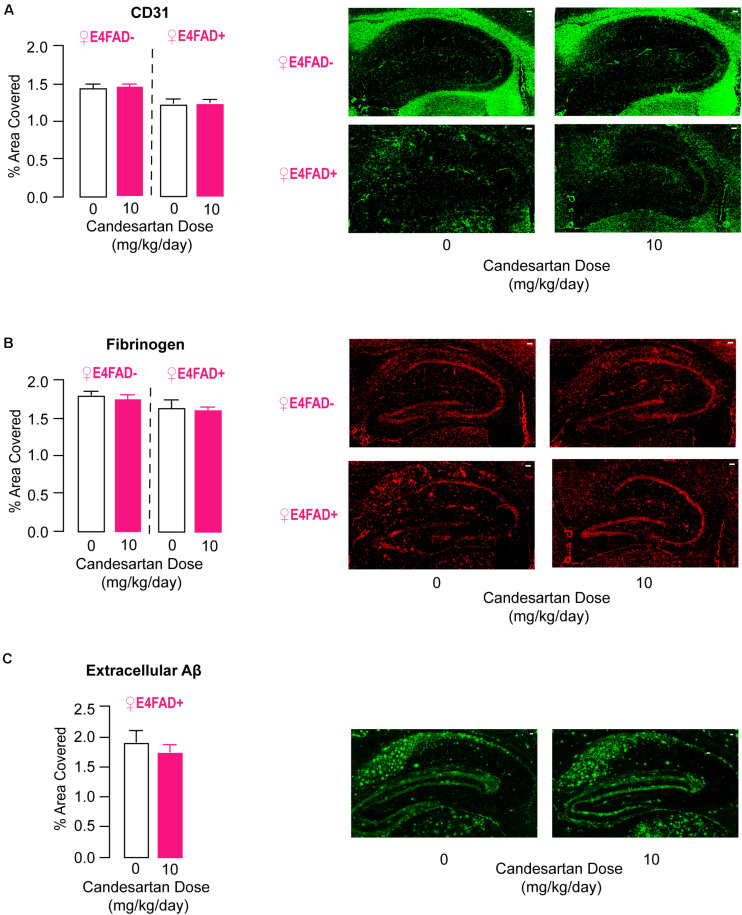
High-dose candesartan treatment did not alter makers of cerebrovascular dysfunction or levels of extracellular Aβ compared to vehicle treatment in female E4FAD− and E4FAD+ mice. In female E4FAD− and E4FAD+ mice treated with 10 mg/kg/day (high dose) candesartan there were no differences in hippocampal area immunostained with **(A)** CD31 [E4FAD−: *t*(16) = 0.28, *p* = 0.78. E4FAD+:*t*(18) = 0.003, *p* = 0.99], **(B)** fibrinogen [E4FAD−: *t*(15) = 0.47, *p* = 0.64. E4FAD+: *t*(14) = 0.23, *p* = 0.82], or **(C)** Aβ [MOAB2 antibody, *t*(18) = 0.74, *p* = 0.47] compared to vehicle when assessed by quantitative IHC analysis. All data expressed as mean +/− SEM. *p* > 0.05 by Student’s *t*-test. Scale bars = 100 μm. See Supplementary Table 2 for details on *n* sizes.

Vessel coverage was measured by staining for the endothelial cell marker CD31, and vascular leakiness was measured by staining for the plasma protein fibrinogen, which does not normally cross an intact cerebrovasculature. There were no differences in CD31 (Figure 5A) or fibrinogen (Figure 5B) staining in the hippocampus of female E4FAD− and E4FAD+ mice treated with high-dose candesartan compared to vehicle, indicating that the beneficial effects of high-dose candesartan treatment, using our treatment regime, occurred independently of effects on the cerebrovasculature.

### High-Dose Candesartan Treatment Did Not Alter Aβ or apoE Levels in Female E4FAD− and E4FAD+ Mice

.5In published studies there is no clear consensus on whether ARB treatment impacts Aβ levels in FAD mice (Wang et al., 2007; Mogi et al., 2008; Takeda et al., 2009; Tsukuda et al., 2009; Danielyan et al., 2010; Ferrington et al., 2011, 2012; Ongali et al., 2014, 2016; Torika et al., 2016, 2017, 2018; Royea et al., 2017; Trigiani et al., 2018). Our data demonstrates that high-dose candesartan treatment was beneficial for memory-relevant behavior, synaptic protein markers, and neuroinflammation in both E4FAD− and E4FAD+ female mice, findings that suggest that these improvements occurred independent of high Aβ levels. However, it was important to assess whether the beneficial effects seen with our treatment design were accompanied by lower Aβ pathology in E4FAD+ mice. Therefore, extracellular Aβ deposits were measured by immunostaining with the anti-Aβ antibody MOAB-2. No alterations in hippocampal MOAB-2 levels were observed in female E4FAD+ mice treated with high-dose candesartan compared to vehicle (Figure 5C). To follow up on this finding, we quantified levels of soluble Aβ42 and total Aβ42 levels by ELISA analysis, since Aβ42 is considered a particularly detrimental form of Aβ (Qiu et al., 2015). There were no differences in hippocampal levels of soluble Aβ42 (Supplementary Figure 4A) or total Aβ42 (Supplementary Figure 4B) in female E4FAD+ mice that were treated with high-dose candesartan compared to vehicle. In addition, in the hippocampus of both female E4FAD− and E4FAD+ mice there were no changes in apoE levels with high-dose candesartan treatment compared to vehicle when assessed by ELISA analysis (Supplementary Figure 4C).

Overall, our data indicate that that beneficial effects of high-dose candesartan treatment in female E4FAD− and E4FAD+ mice occurred independently of altering Aβ and apoE levels.

## Discussion

In this study we have demonstrated that systemic treatment with candesartan cilexetil results in improved short-term memory, higher synaptic protein levels, and modulated neuroinflammatory markers in female mice, but not male mice, that express the human *APOE4* gene. These data support further research on the role of the AT1 receptor in modulating neuroinflammation and neuronal function from mechanistic and therapeutic perspectives.

### Clinical and *in vivo* AD Relevance for the Modulation of Behavior by ARB Treatment

Prospective cohort analyses and observational studies support that ARBs could limit the detrimental effects of hypertension on AD risk and progression (Tzourio, 2007; Li et al., 2010; Davies et al., 2011; Kume et al., 2012; Ho et al., 2017). For example, the use of ARBs is associated with lower risk of developing AD (Li et al., 2010), slower cognitive decline (Kume et al., 2012), and improvements in short- and long-term memory preservation in AD patients (Ho et al., 2017); however, there are some contrasting data (Hsu et al., 2013; Kurinami et al., 2013). Underlying the conflictions in human data could be the age of hypertension onset in AD patients. Indeed, increasing evidence supports that hypertension in mid-life is particularly harmful for AD risk and progression, and therefore ARB treatment should be administered early (Launer et al., 2000). Importantly, through these initial studies a key concept emerged: that ARBs could modulate AD-relevant pathology independent of hypertension. Human evidence for this concept is that ARB use, as compared to the use of other classes of antihypertensive medications such as ACE inhibitors and Beta-blockers, is associated with lower neuritic plaque count and a reduction in neurofibrillary tangles in AD patient brain tissue (Hoffman et al., 2009; Hajjar et al., 2012). In addition, the AT1 receptor is expressed in brain regions relevant to learning, memory, and AD (e.g., hippocampus and cerebral cortex) in mice (Allen et al., 1998), rats (Lenkei et al., 1997), gerbils (Tonelli et al., 2000), dogs (Speth et al., 1985), and humans (MacGregor et al., 1995). Further, higher levels of AT1 receptor activation can result in memory impairment and neuronal dysfunction (Benicky et al., 2009; Saavedra, 2012a,b, 2016). The collective evidence that AT1 receptor activation could contribute to changes in learning, memory, and synaptic biology relavent for AD, led to an evaluation of ARB activity in AD-relevant rodent models. Most of these studies support that ARB treatment is beneficial for memory-relevant behavior and markers of neuronal function in FAD mouse models that overexpress human Aβ. For instance, telmisartan (Mogi et al., 2008; Tsukuda et al., 2009; Torika et al., 2017) losartan (Danielyan et al., 2010; Ongali et al., 2014; Royea et al., 2017), valsartan (Wang et al., 2007), and candesartan (Trigiani et al., 2018) treatment resulted in improvements in learning and memory-type behavior in various FAD mouse models (e.g., APP/PS1, J20, and 5xFAD). Further, olmesartan treatment reversed deficits in hippocampal LTP in Aβ-injected mice (Takeda et al., 2009), candesartan treatment resulted in higher dendritic arboriztion in J20 mice (Trigiani et al., 2018), and losartan treatment resulted in higher tyrosine hydroxylase expression in APP/PS1 mice (Danielyan et al., 2010). To date, eprosartan is the only ARB tested that appeas not to modulate memory-relevant behavior in 3xTG AD mice (Ferrington et al., 2012) and in APP/PS1 mice (Wiesmann et al., 2017). However, this could be because eprosartan has lower bioavailability and potency than other clinically used AT1 receptor antagonists, and therefore could require higher doses for biological activity in FAD mice (Michel et al., 2013). Further, the choice of prodrug (candesartan cilexetil) used also impacts drug pharmacokinetics and pharmacodynamics. Our data are in agreement with and expand upon reports indicating beneficial effects of ARBs on memory and markers of neuronal function in AD-relevant rodent models. In our current study, candesartan improved memory-relevant behavior and presynaptic protein levels in mice in the context of two key AD risk factors: female sex and *APOE4* (see below for detailed discussion). Therefore, collectively there is a growing body of human and *in vivo* evidence supporting that ARBs may have therapeutic value in AD.

### Inflammatory Processes Modulated by ARB Treatment

Key for the full evaluation of ARBs as an AD therapy is understanding their cellular mechanism(s) of action. Although precise mechanisms cannot be identified with our study design, our data on changes in AD-relevant pathology could help guide future mechanistic research on this topic. There are a number of potential explanations for our data, however, we would like to initially focus on a speculative mechanism of action whereby candesartan blocks the AT1 receptor on astrocytes or microglia, resulting in altered cellular phenotypes that are beneficial and/or less detrimental for neuronal function and behavior. This proposed mechanism raises important points regarding candesartan brain bioavailability, angiotensin II and AT1 receptor levels/expression and functions in the brain, and neuroinflammatory phenotypes linked to neuron function.

Angiotensin receptor blockers were originally developed for the treatment of hypertension, and therefore no pharmacokinetic studies directly centered on brain bioavailability have been conducted, which is also a limitation of our study (see “limitations” section below). However, there is indirect evidence that candesartan can cross the blood-brain barrier following peripheral administration. Candesartan when administered via osmotic minipumps at 0.1, 0.5, and 1.0 mg/kg/day inhibited AT1 receptors in the brain when assessed by autoradiography studies in wild type rats (Nishimura et al., 2000). Similarly, candesartan administered via i.v. at doses of 0.01, 0.1, 1, or 10 mg/kg/day blocked angiotensin II–induced (i.c.v.) drinking and pressor responses in rats (Gohlke et al., 2002). Thus, candesartan may be brain penetrant in wild type rodents when administered peripherally, and data also suggests that this is the case for AD-relevant rodent models. When administered in drinking water at a dose of 10 mg/kg/day, candesartan modulated neuroinflammation and hippocampal dendritic arborization in J20 mice (Trigiani et al., 2018). In general, there are a number of properties that determine the extent that a drug is brain penetrant from both a chemical and biological perspective. Two important biological aspects relate to the specialized functions of the blood-brain barrier: low paracellular permeability and high expression of efflux transporters. Regarding the former, research, including our own (Tai et al., 2017), has demonstrated that there is higher paracellular permeability and blood-brain barrier breakdown in mice that express *APOE4* at the ages used in this study. Further, although the AT1 receptor is expressed on brain endothelial cells and excessive AT1 receptor signaling has been shown to directly impair endothelial cell function (Fleegal-DeMotta et al., 2009), in our study candesartan treatment did not result in changes to vascular permeability (fibrinogen) in female E4FAD− and E4FAD+ mice. Therefore, blood-brain barrier dysfunction may be too far advanced in E4FAD mice at the ages evaluated in this study (Thomas et al., 2016, 2017; Zaldua et al., 2020) for candesartan to have exerted a beneficial effect, or the contribution of the AT1 receptor to cerebrovascular dysfunction is minimal in E4FAD mice. For either event, the result may be that a disrupted blood-brain barrier in E4FAD mice enabled candesartan to cross into the brain. Future studies focused on detailed pharmacokinetic analysis of candesartan are important for a mechanistic interpretation of how systemic treatment improved behavioral function in female *APOE4* mice.

Once inside the brain our working hypothesis is that candesartan prevents angiotensin II from activating AT1 receptors on glia, which raises the important discussion points of the source and levels of angiotensin II in the brain under normal and pathological conditions, if glia respond to angiotensin II, and whether blocking the AT1 receptor modulates neuroinflammation. One way that angiotensin II could enter the brain is by crossing in from the periphery through receptor mediated transport, diffusion, and/or at the circumventricular organs. Alternatively, there may be local production and it has been proposed that cells within the brain collectively express proteins and enzymes to enable angiotensin II production (McKinley et al., 2003; Sakai et al., 2007). For either scenario, the physiological functions of angiotensin II in the brain, and specifically in the hippocampus, are unclear, but could relate to communicating blood-pressure responses from the periphery to the brain, or an adaptation of angiotensin II to act as a local homeostatic signaling molecule related to inflammatory or stress stimuli. During aging, with *APOE4*, female sex, and/or Aβ pathology the amount of angiotensin II may be modulated in the brain to levels that become detrimental. For example, a disrupted cerebrovasculature would enable higher peripheral entry of angiotensin II, and local brain production could also increase in response to aging and AD-relevant stressors. Thus, although in the current study we found no change in angiotensin II levels with candesartan treatment in female E4FAD− or E4FAD+ mice, the possibility remains that angiotensin II levels increase with aging to activate glia. Alternatively, rather than levels of angiotensin II, in female E4FAD− and E4FAD+ mice there may be a synergistic interaction between AT1 receptor signaling and other signaling pathways modulated by *APOE4* and female sex in glia. Like angiotensin II, the extent that the expression of the AT1 receptor is regulated in the brain is unclear. In our study there were no differences in levels of the AT1 receptor in hippocampal brain homogenates between vehicle and candesartan-treated mice, but the way that we collected tissue for IHC analysis precluded cell-type specific evaluation of AT1 receptor expression and levels (non-fixed perfused). However, in general, other research groups have identified that glia express the AT1 receptor, that glial AT1 receptors respond to angiotensin II, and/or that blocking the AT1 receptor in glia modulates inflammation. In astrocytes, AT1 receptor expression has been confirmed by quantitative IHC analysis in adult wild type rats (Fogarty and Matute, 2001) and mouse models of multiple sclerosis (Lanz et al., 2010). In addition, angiotensin II treatment of astrocytes results in superoxide production and senescence (Liu et al., 2011), and data from *in vivo* lesion studies support that AT1 receptor activation in astrocytes promotes a neuroinflammatory phenotype (Fuchtbauer et al., 2011). Studies have also demonstrated that microglia express the AT1 receptor in the cerebral cortex of adult rats (Wu et al., 2013) and mice (Phipps et al., 2018; Cui et al., 2019), angiotensin II activates microglia *in vitro* (Villar-Cheda et al., 2012), and blocking microglial AT1 receptor suppresses their activation in response to inflammatory stimuli (Benicky et al., 2011; Sun et al., 2015). Further, ARB treatment of FAD rodent models lowers markers of astrocyte and microglia activation as we found in E4FAD mice (Danielyan et al., 2010; Ongali et al., 2014; Torika et al., 2016; Royea et al., 2017; Torika et al., 2017, 2018; Trigiani et al., 2018). There are many questions remaining surrounding the levels and functions of angiotensin II and glial expressed AT1 receptors in physiological and pathological states (see “limitations” section). However, the studies described above suggest that glia express the AT1 receptor which, when activated, modulates inflammatory responses. Thus, it is possible that during aging, AD-relevant risk factors and stressors (e.g., *APOE4*, Aβ) disrupt normal homeostatic functions of AT1 receptor signaling in glia, which underlies the mechanisms of how ARB treatment improves behavior.

The general concept that blocking the AT1 receptor on glia can alter their activation states, raises the more specific question of how this resulted in improved behavior with candesartan treatment. Although hippocampal levels of Iba1, but not GFAP, correlated with improvements in memory-relevant behavior in female E4FAD mice, there are limitations in this type of analysis (e.g., assumptions in linearity, read-out specificity, and lack of longitudinal analysis). Therefore, we will discuss in more detail ways that blocking the AT1 receptor in astrocytes or microglia could have improved neuronal function in female E4FAD mice. Astrocytes perform multiple functions to maintain homeostasis of the central nervous system including buffering potassium ions, recycling neurotransmitters, regulating homeostasis of lactate/glucose, buffering pH, aiding interstitial fluid bulk flow, secreting protective molecules for neurons and the vasculature, as well as modulating cytokine and chemokine production. The idea that any of these astrocytic functions are disrupted in AD or FAD mice was initially based on GFAP staining, as when activated the levels of GFAP (intermediate filaments prominent in processes) are higher in astrocytes. In FAD models and AD human postmortem tissue, GFAP immunostaining is higher than wild-type or age-matched controls, respectively, particularly surrounding Aβ extracellular deposits and neurofibrillary tangles of tau (Simpson et al., 2010; Verkhratsky et al., 2010). *APOE4* is also associated with higher GFAP levels in FAD mice and in response to inflammatory stress (Zhu et al., 2012; Fernandez et al., 2019). The higher GFAP immunoreactivity in AD-relevant contexts likely represents the amount of astrocytes that have become active, rather than a change in absolute numbers or migration. Thus, GFAP in many ways is a surrogate marker for astrocytic phenotypes, but the link to functional consequences is less clear and becoming increasingly complicated. Indeed, single cell gene expression and transcription analysis studies continue to identify several different astrocytic phenotypes including A1/A2, disease associated (Habib et al., 2020), proinflammatory, neurotoxic, as well as associated sub-sets (reviewed in Spanos and Liddelow, 2020). In many cases the gene profiles across studies at least partially overlap, and more research is required to synergize nomenclature across studies and identify the functions of different astrocytic phenotypes. Nonetheless, there is an apparent consensus across the transcriptomic studies that in a chronic condition such as AD, it is important for astrocytes to modulate inflammatory phenotypes (i.e., prevention of neurotoxicity induced by cytokines) while promoting homeostatic functions [neuroprotective functions described above (Spanos and Liddelow, 2020)], which may have been the case in candesartan-treated female E4FAD mice. Hopefully more specific and tangible concepts will evolve as studies on astrocyte biology begin to incorporate how treatments modify their transcriptomics phenotypes. Like astrocytes, the functions of microglia in the brain are complex and they exist as a heterogenous phenotype. In general, the functions of microglia include phagocytosis of apoptotic cells, pathogens, Aβ, and synapses/synaptic pruning, and secretion of cytokines/chemokines or protective factors. In FAD mice and AD patients, there are higher numbers of Iba1-positive cells, which likely represents migration of microglia to the area, or potentially infiltration and differentiation of peripheral immune cells (Hein and O’Banion, 2009; Balducci et al., 2018; Toscano et al., 2020). In addition to number, the activation of microglia is associated with a morphological shift in appearance from small cell somas and long processes to large cell bodies with fewer and shorter processes (Schlachetzki and Hull, 2009). Qualitatively we did not observe any differences in the morphology of microglia with candesartan treatment in E4FAD mice, and therefore it is the number of activated microglia that was likely modulated by candesartan treatment. However, a lack of morphological change in glial cells does not necessarily imply a lack of an impact on phenotypes. Indeed, single cell transcriptomic analysis has identified different types of microglial phenotypes in FAD mice (Keren-Shaul et al., 2017; Habib et al., 2020), in neurodegenerative disorders, and it has also been proposed that *APOE* genotype modulates microglial phenotypes. However, when identified by gene expression profiling, whether a disease associated microglial phenotype is beneficial or detrimental for neuronal function is still subject for debate. For example, *APOE4* has been proposed to be associated with a disease associated transcriptomic phenotype, which functionally may manifest as higher cytokine production, impaired migration, and lower phagocytosis (Fernandez et al., 2019). At the same time, other gene expression profiles described as disease associated are interpreted as beneficial. For example, it has been suggested that unblocking microglial specific check points to enrich subsets of disease associated microglia is optimal for promoting neuron function (Keren-Shaul et al., 2017). In our study, we saw lower Iba1 staining with candesartan treatment and no change in Aβ levels, which would be characteristic of a beneficial phagocytotic phenotype. Therefore, our data suggest that ARBs lowered the amount of activated microglia, consistent with other ARB treatment studies in FAD mice (Danielyan et al., 2010; Ongali et al., 2014; Torika et al., 2016, 2017, 2018; Royea et al., 2017; Trigiani et al., 2018), although it is possible the remaining microglia were of a beneficial phenotype. Overall, astrocytes and microglia are linked to multiple functions that could have been modulated by ARB treatment in female E4FAD− and E4FAD+ mice. Traditionally, shifting cytokine and chemokine levels/phenotypes has been considered the major beneficial consequence of lower GFAP and/or Iba1 levels. However, candesartan treatment did not result in changes in levels of many common cytokines/chemokines in the hippocampus of female E4FAD− or E4FAD+ mice. There are caveats to these data including: for cytokines/chemokine levels the effect of candesartan could be acute (i.e., influenced by amount of drug onboard at time of sacrifice), cytokines/chemokines that we did not measure were altered, and/or there were hippocampal sub-region differences in cytokine/chemokine levels. Yet, our data may be more consistent with the idea that candesartan modulated other glial functions in order to improve neuron function, such as homeostatic regulation of potassium ions, neurotransmitters, and energy supply, secretion of neuroprotective factors, and surveillance/phagocytosis. Future studies using single-cell transcriptomics analysis could reveal the extent that candesartan alters astrocytic and microglial phenotypes in E4FAD mice, which combined with proteomics and functional assays for neurons, could reveal more detailed mechanisms of how AT1 receptor activation in glia impacts neuron function and behavior.

Neuroinflammation is a complex and at times abstract concept that refers to activation states and phenotypes of multiple cell types (astrocytes, microglia, pericytes, endothelial cells, neurons, and peripheral immune cells). In the context of our study, we found changes in the levels of astrocytes and microglia, however, there are alternative hypotheses for how candesartan could have improved behavior and neuroinflammation in our study. Candesartan may have improved behavior in *APOE4* mice through directly lowering AT1 receptor activation in neurons (Sandgren et al., 2018). Indeed, angiotensin II has been demonstrated to modulate synaptic proteins levels *in vitro* (Kurihara et al., 2008) and regulate neuronal firing rates (Li et al., 2003; Li and Pan, 2005) and membrane ionic currents when assessed using electrophysiological analysis (Sumners et al., 1996; Wang et al., 1997). In addition, blocking AT1 receptor signaling on neurons could have also lowered glial activation (Elsaafien et al., 2020) which in turn reduces neuronal dysfunction. Alternatively, the beneficial effects of ARBs on behavior could be mediated through modulation of inflammatory processes related to AT1 receptor signaling in the periphery. The AT1 receptor is expressed by multiple cell types related to control of peripheral inflammation (e.g., macrophages) and blood pressure (e.g., smooth muscle cells, endothelial cells; Villapol and Saavedra, 2015). Often these two processes are considered linked, since ARB treatment has general anti-inflammatory properties in both humans with hypertension (Fliser et al., 2004) and *in vivo* (Marchesi et al., 2008). However, recent studies support that ARBs can alter peripheral inflammation independent of blood pressure-lowering effects in hypertension (Pang et al., 2012; Villapol and Saavedra, 2015). Peripheral inflammation, such as changes in cytokine and chemokine levels as well as activated immune cells, is linked to AD-relevant pathology and behavior in AD-relevant mouse models including with *APOE4* (Benicky et al., 2009; Marottoli et al., 2017). Indeed, with *APOE4* there is an altered inflammatory response in both humans and mice treated with inflammatory stressors (Gale et al., 2014; Tai et al., 2015). Therefore, one potential explanation for our data is that ARBs modulated peripheral inflammation to improve behavior and synaptic function. However, altering peripheral inflammation *in vivo* is typically associated with improvements in cerebrovascular function, which we did not observe in this study. An additional explanation is that there is a connection between the lower blood pressure in female E4FAD− and E4FAD+ mice after ARB treatment and improved behavioral function. A link between lowering blood pressure in non-hypertensive mice and improved memory has not been established, and we observed lower blood pressure in male E4FAD mice without an associated improvement in behavior. Further, previous studies evaluating the activity of ARBs in FAD mice have found similar memory-improving benefits in the absence of any changes to systemic blood pressure (Wang et al., 2007; Mogi et al., 2008; Danielyan et al., 2010; Ongali et al., 2014; Royea et al., 2017), suggesting that ARBs modulate learning and memory-type behavior independent of blood pressure. Inducing hypotension in a non-hypertensive AD patient is also likely to be considered a detrimental side effect. In addition, *APOE4* is associated with several other peripheral changes such as alterations in metabolism, and the gut microbiome, any of which could have been modulated by ARB treatment (Gregg et al., 1986; Bandaru et al., 2009; Maldonado Weng et al., 2019; Parikh et al., 2020). Taken together, further research could aid in evaluating the extent that the AT1 receptor in the periphery modulates inflammation in AD-relevant contexts and the development of novel ARBs that display higher brain penetration could limit clinical hypotensive effects of ARBs.

### Role of *APOE4*, Female Sex, and Aβ in Contributing to ARB Activity

Our study raised several important discussion points: that candesartan benefited memory-relevant behavior in female, but not male, mice expressing *APOE4*, that these benefits occurred both in the presence (E4FAD+) and absence (E4FAD−) of Aβ overproduction, and that beneficial effects on behavior with candesartan treatment coincided with alterations in gliosis and synaptic protein levels in the hippocampus of female mice.

In general, human data support the concept that *APOE4* and female sex interact to exacerbate AD risk and pathology. For example, *APOE4* confers a greater lifetime risk of AD and accelerated degeneration rates in females compared to males (Payami et al., 1994; Farrer et al., 1997; Riedel et al., 2016). The changes that result in this increased risk are typically thought to occur post-menopause, however, *in vivo* studies demonstrate that *APOE4*-female sex interactions occur independent of changes in sex hormones (Grootendorst et al., 2005; Bour et al., 2008; Leung et al., 2012; Siegel et al., 2012; Segev et al., 2013; Tai et al., 2017). Although there are multiple caveats, in our study design we attempted to select ages that matched AD-relevant pathology by treating male mice at a later timepoint (8–12 months of age) than female mice (6–10 months of age). Thus, one potential explanation for our data is that candesartan targets dysfunctional pathways that contribute to altered neuron function to a greater extent in females than in males that express *APOE4*; one such pathway could be neuroinflammation. Although we did not perform analysis of inflammation on tissue from male E4FAD− and E4FAD+ mice (we encountered a technical issue that resulted in sample spoiling), published data support this concept as in general, females experience earlier and more aggressive neuroinflammation with age than males (Hanamsagar and Bilbo, 2016; Spychala et al., 2017; Doran et al., 2019), and female E4FAD+ mice have higher hippocampal and cortical astrogliosis compared to E3FAD+ mice (Balu et al., 2019; Stephen et al., 2019). Therefore, female mice that express *APOE4* may be more susceptible to neuroinflammation-associated memory dysfunction with age. Another potential pathway underlying the differences in treatment effects by sex, is that angiotensin II levels and/or AT1 receptor activation are higher in *APOE4* females than males. While sexual dimorphisms in angiotensin II metabolism, angiotensin receptor expression levels, and angiotensin receptor activation have been reported in the periphery (Thomas et al., 2016, 2017; Zaldua et al., 2020), there is limited data available regarding the effects of biological sex on brain angiotensin peptide and receptor levels. Although in the present study, treatment did not alter angiotensin peptide or receptor levels, a focus of our ongoing studies is identification of the extent that the angiotensin II/AT1 receptor axis is differentially influenced by age, sex, Aβ, and *APOE* genotype.

Recent evidence supports the idea that regardless of high human Aβ levels, *APOE4* is sufficient to cause behavioral and neuronal dysfunction in female mice (Sullivan, 2008; Leete et al., 2018). In fact, our results suggest that ARBs have the potential to mitigate *APOE4*-associted deficits in female mice even in the absence of Aβ overproduction and that candesartan may be acting through Aβ-independent pathways in the brain. That we saw identical treatment benefits in both E4FAD− and E4FAD+ female mice and that we did not observe any lowering of Aβ levels after treatment in E4FAD+ mice, also brings up an important point regarding the overall effect of ARBs on Aβ pathology, of which there are contrasting data. Indeed, while some studies in FAD mice have reported profound reductions in Aβ levels with ARB treatment (Wang et al., 2007; Mogi et al., 2008; Danielyan et al., 2010; Torika et al., 2016, 2017, 2018), others have shown no effects (Mogi et al., 2008; Ferrington et al., 2011, 2012; Ongali et al., 2014; Royea et al., 2017; Trigiani et al., 2018; Royea et al., 2020). These discrepancies may be related to a secondary property of certain ARBs: activation of Peroxisome Proliferator- Activated Receptor γ (PPARγ). PPARγ is a nuclear hormone receptor that plays an important role in fatty acid storage, glucose metabolism, and inflammation. Importantly, nuclear receptor agonists (Tai et al., 2014a; Koster et al., 2017; Moutinho et al., 2019) including for PPARγ (Camacho et al., 2004), lower Aβ levels in FAD mice and increase Aβ clearance in cellular models *in vitro*. Therefore, since different ARBs have different PPARγ-activating properties, this could explain why some studies found robust Aβ effects following ARB treatment while others did not. Ultimately, combined with the results of previous studies, our data suggest that ARB treatment in FAD mice modulates learning and memory-type behavior, synaptic function, and neuroinflammation, independent of alterations in Aβ levels, and in the absence of Aβ overproduction altogether (E4FAD- mice). However, we cannot eliminate the prospect that candesartan treatment in E4FAD+ mice partially restored behavioral and synaptic deficits induced by Aβ.

*APOE4* is linked to alterations in neuronal function throughout the brain; however, dysfunctions in the hippocampus are particularly important in the context of learning and memory-type behavior. Indeed, our data suggest that the effects of candesartan in female E4FAD− and E4FAD+ mice were localized primarily to the hippocampus as this is where reductions in glial cell number/activation and improvements in synaptic protein levels were observed. The cerebral cortex is another region of the brain that is closely linked to memory and cognition; however, in the current study, candesartan did not appear to modulate synaptic protein levels or gliosis in the cortex of female E4FAD+ mice. One potential explanation for the lack of effects in the cortex, is that changes were concentrated only in certain cortical subregions, for example the prefrontal cortex, or in certain cortical layers. Alternatively, concentrations of the AT1 receptor may differ by brain region in E4FAD mice, with higher concentrations present on cells in the hippocampus as compared to the cortex. While it is well established that the distribution of the AT1 receptor is heterogeneous in the brains of wild type mice (Allen et al., 1998) and rats (Lenkei et al., 1997), no such localization studies have been conducted in FAD rodents. It is therefore possible that the relative distribution of the AT1 receptor by brain region is differentially affected by biological sex, Aβ, and/or *APOE* genotype. There are several additional explanations such as brain region differences in candesartan pharmacokinetics, variations in the influence of inflammation on synaptic function by brain region, and changes with candesartan treatment for neuroinflammatory and synaptic readouts differed from those that we measured in this study.

### Limitations

Overall, due to the nature of our study design there are limitations on the type of mechanistic and therapeutic insights that we can provide surrounding the applicability of ARBs for the treatment of AD. One issue is that we did not identify the precise cellular functional mechanism(s) that underlie the beneficial effects of candesartan in mice that express human *APOE4*. Although suggested by our correlative data, we are unable to draw the strict conclusion that candesartan improves memory and synaptic function by modulating neuroinflammation in E4FAD female mice. In addition, our study has raised an additional set of key questions including: Why was ARB treatment beneficial in female and not male mice that express human *APOE4*? What is the optimal treatment age, dose, and duration for ARB treatment of E4FAD mice? Would ARBs be beneficial in mice that express *APOE3*? Is candesartan the optimal therapeutic candidate? What is the endogenous function of brain AT1 receptor signaling in physiological conditions and during neurodegeneration? To help address these issues our current research is focused on understanding how *APOE* genotype, Aβ levels, age, and sex interact to modulate the angiotensin II/AT1 receptor axis in different brain regions of EFAD mice in relation to neuroinflammation and altered behavioral function. Such an understanding will form the basis for further mechanistic (e.g., identify the source of angiotensin II in the brain along with the functional effects of cell-type specific AT1 receptor knockdown) and preclinical therapeutic studies (e.g., vary age of treatment onset, duration of treatment, and route of administration). Finally, a limitation of the current study is that we restricted analysis to candesartan. Therefore, from a therapeutic standpoint, detailed preclinical ARB treatment studies are required with in-depth pharmacokinetic analyses in order to determine whether any of the currently available ARBs are optimal candidates for the treatment of AD, or if there may be more therapeutic potential in developing a new ARB that is optimized for PPARγ activating properties and brain penetrance. Addressing these questions are fundamental next steps for translating findings from ARB treatment studies in AD-relevant rodent models into therapeutic strategies for human AD patients.

## Summary

The goal of this study was to evaluate the activity of long-term ARB treatment at modulating AD-relevant pathology in mice that express human *APOE4*. Our data demonstrate that ARB treatment is beneficial for memory-relevant behavior, hippocampal synaptic protein levels, and neuroinflammation in female mice that express *APOE4* both in the presence (E4FAD+) and absence (E4FAD−) of high Aβ levels. Thus, development of therapies targeting the angiotensin II/AT1 receptor axis could provide options for neuroinflammation in female *APOE4* carriers.

## Data Availability Statement

The raw data supporting the conclusions of this article will be made available by the authors, without undue reservation.

## Ethics Statement

The animal study was reviewed and approved by UIC Institutional Animal Care and Use Committee.

## Author Contributions

LMT, SBS, and GRJT conceived and designed the experiments, performed the experiments, analyzed and interpreted the data, and wrote the manuscript. SZ, AD, KK, KD, and FMM performed the experiments. All authors contributed to the article and approved the submitted version.

## Conflict of Interest

GRJT was an inventor on patents owned by UIC. The remaining authors declare that the research was conducted in the absence of any commercial or financial relationships that could be construed as a potential conflict of interest.
