# Supplementary material for: Systemic Candesartan Treatment Modulates Behavior, Synaptic Protein Levels, and Neuroinflammation in Female Mice That Express Human APOE4
Source: Front Neurosci. 2021 Feb 10;15:628403. doi: 10.3389/fnins.2021.628403 (PMC7902885; doi:10.3389/fnins.2021.628403)
Supplement: Supplementary file 1 [file Data_Sheet_1.PDF]

Supplementary Material

1 Supplementary Figures

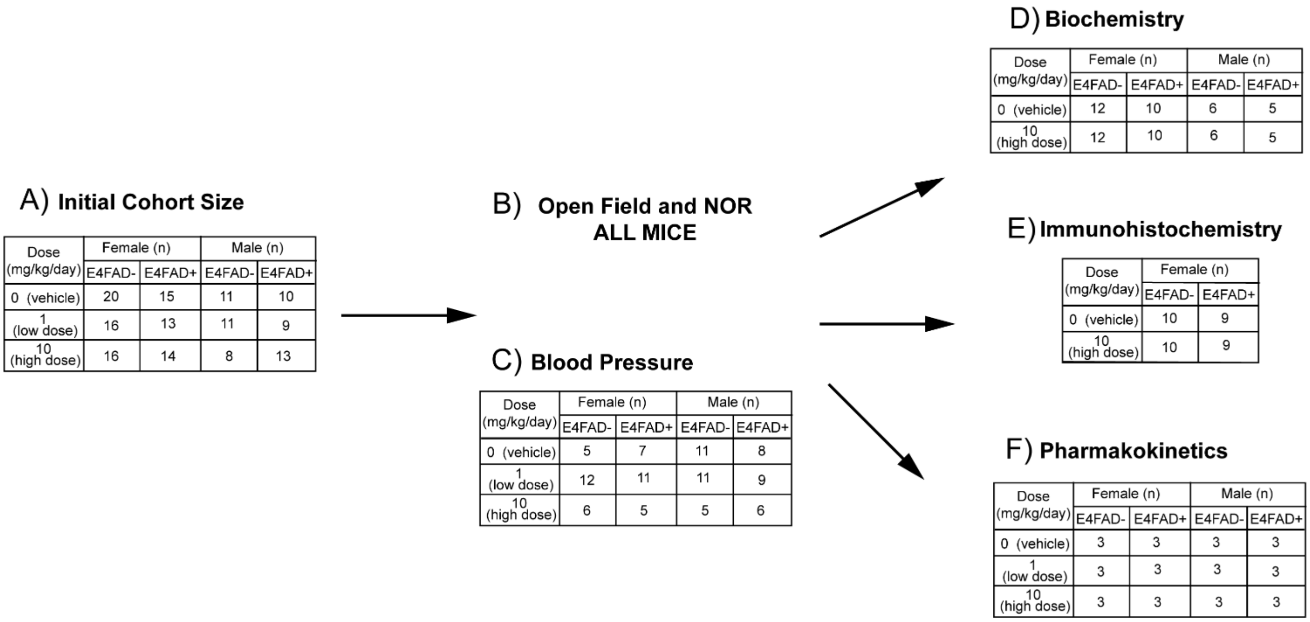

A) Initial Cohort Size

Dose (mg/kg/day)	Female (n)		Male (n)	
	E4FAD-	E4FAD+	E4FAD-	E4FAD+
0 (vehicle)	20	15	11	10
1 (low dose)	16	13	11	9
10 (high dose)	16	14	8	13

C) Blood Pressure

Dose (mg/kg/day)	Female (n)		Male (n)	
	E4FAD-	E4FAD+	E4FAD-	E4FAD+
0 (vehicle)	5	7	11	8
1 (low dose)	12	11	11	9
10 (high dose)	6	5	5	6

D) Biochemistry

Dose (mg/kg/day)	Female (n)		Male (n)	
	E4FAD-	E4FAD+	E4FAD-	E4FAD+
0 (vehicle)	12	10	6	5
10 (high dose)	12	10	6	5

E) Immunohistochemistry

Dose (mg/kg/day)	Female (n)	
	E4FAD-	E4FAD+
0 (vehicle)	10	9
10 (high dose)	10	9

F) Pharmakokinetics

Dose (mg/kg/day)	Female (n)		Male (n)	
	E4FAD-	E4FAD+	E4FAD-	E4FAD+
0 (vehicle)	3	3	3	3
1 (low dose)	3	3	3	3
10 (high dose)	3	3	3	3

Supplementary Figure 1. Study Design. **(A)** Female and male mice that express human *APOE4* in the absence (E4FAD-) and presence (E4FAD+) of FAD mutations were treated with either vehicle (0mg/kg/day), 1mg/kg/day candesartan, or 10mg/kg/day candesartan. **(B)** All mice, regardless of treatment condition, underwent behavioral testing (open field and novel object recognition) prior to sacrifice. 12 mice (4x Female E4FAD-, 3x Female E4FAD+, 3x Male E4FAD-, 2x Male E4FAD+) were eliminated from NOR analysis because they failed to meet the inclusion criteria of 20 seconds total object investigation time. **(C)** A subset of female and male mice from all treatment conditions had blood pressure measured following behavioral testing. As directed by the data, **(D)** biochemical analysis and **(E)** immunohistochemical analysis was conducted in a subset of E4FAD- and E4FAD+ mice only from the vehicle and 10mg/kg/day conditions. **(F)** Finally, pharmacokinetic analysis was conducted on a subset of mice from all experimental conditions to determine plasma drug concentrations.

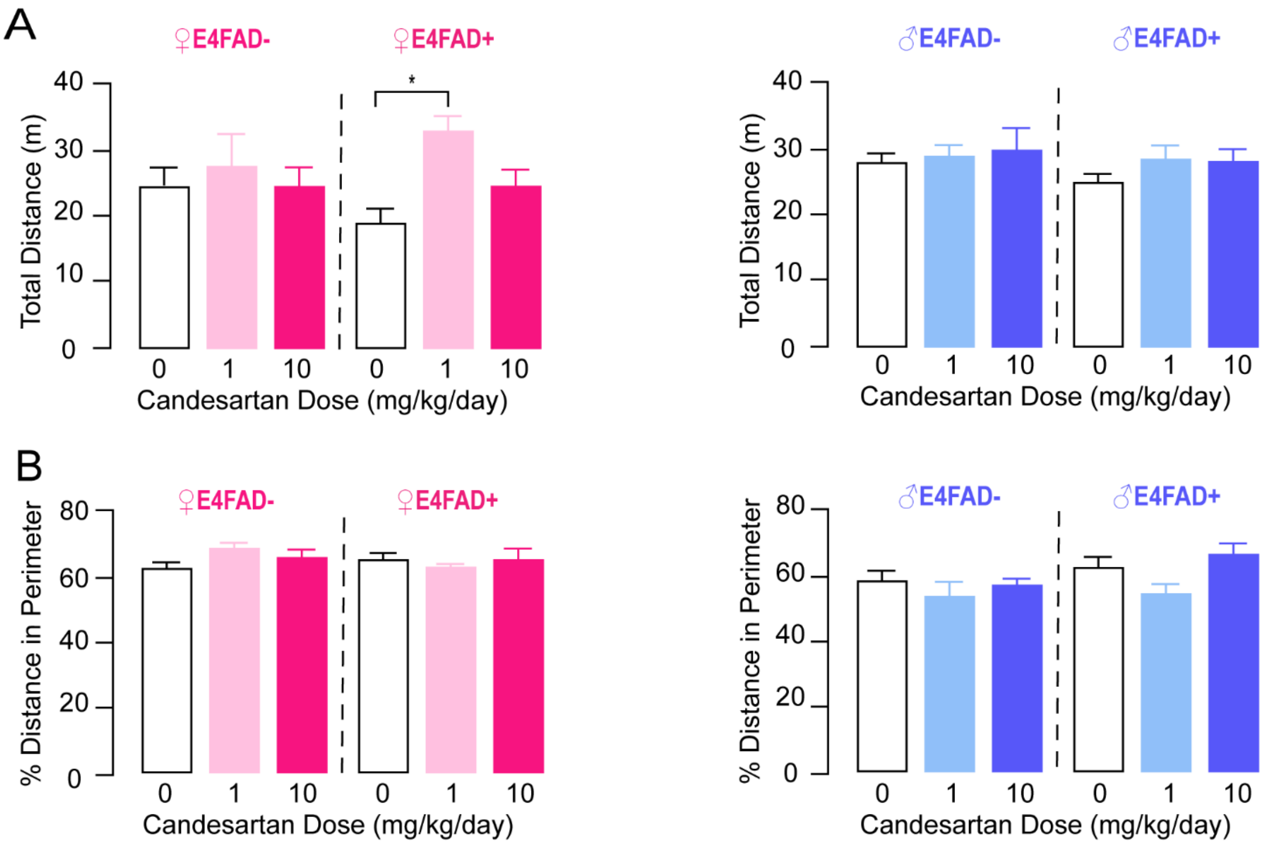

Supplementary Figure 2. Candesartan treatment had no effect on locomotion and anxiety-like behavior compared to vehicle treatment in female and male E4FAD- and E4FAD+ mice. (A) Compared to vehicle treatment (0mg/kg/day), there were no differences in total distance travelled with 1mg/kg/day candesartan (low dose) and 10mg/kg/day candesartan (high dose) treatment in female and male E4FAD- and E4FAD+ mice (Female E4FAD-: $F(2,21)=0.11$, $p=0.89$. Male E4FAD-: $F(2,30)=1.19$, $p=0.32$. Male E4FAD+: $F(2,26)=0.16$, $p=0.85$). The exception was for female E4FAD+, where mice treated with the low dose of candesartan traveled farther than mice treated with vehicle ($F(2, 37)=7.19$, $p=0.0023$). **(B)** Compared to vehicle treatment, there were no differences in % distance traveled in perimeter of open field with 1mg/kg/day candesartan (low dose) and 10mg/kg/day candesartan (high dose) treatment in female and male E4FAD- and E4FAD+ mice (Female E4FAD-: $F(2,22)=0.05$, $p=0.95$. Female E4FAD+: $F(2,36)=1.71$, $p=0.19$. Male E4FAD-: $F(2,30)=0.50$, $p=0.61$) compared to vehicle. Note: male E4FAD+ mice treated with the 1mg/kg/day candesartan spent more time in the perimeter than mice treated with the 10mg/kg/day candesartan ($F(2,26)=3.69$, $p=0.039$), but neither differed significantly from mice treated with vehicle. All data expressed as mean +/- SEM. $p>0.05$ by one-way ANOVA and Dunnet's post-hoc analysis for candesartan dose comparisons to vehicle. See Supplementary Table 2 for details on *n* sizes.

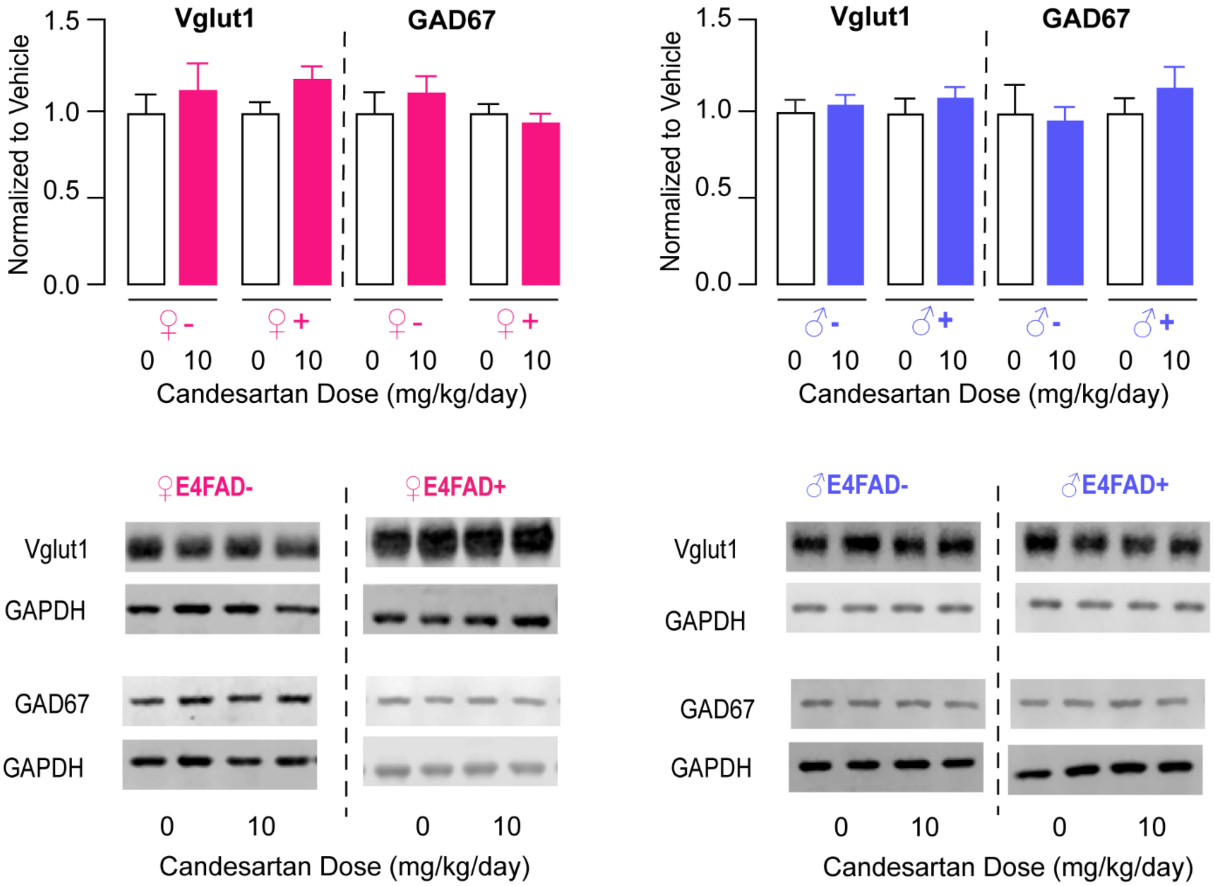

Supplementary Figure 3. High-dose candesartan treatment did not alter hippocampal levels of Vglut1 or GAD67 compared to vehicle treatment in female and male E4FAD- and E4FAD+ mice. There were no differences in hippocampal levels of Vglut1 or GAD67 in female and male E4FAD- and E4FAD+ mice treated with 10mg/kg/day candesartan (high dose) compared to vehicle (Female E4FAD-: $t(10)=0.25$, $p>0.05$. Female E4FAD+:$t(9)=0.56$, $p>0.05$. Male E4FAD-: $t(9)=0.43$, $p>0.05$. Male E4FAD+: $t(9)=0.65$, $p>0.05$), or GAD67 (Female E4FAD-: $t(10)=0.43$, $p>0.05$. Female E4FAD+: $t(9)=0.66$, $p>0.05$. Male E4FAD-: $t(10)=0.25$, $p>0.05$. Male E4FAD+: $t(9)=0.65$, $p>0.05$) . Quantification of each protein was normalized to GAPDH as a loading control and all data are expressed as a ratio to vehicle-treated mice. All data expressed as mean +/- SEM. $p>0.05$ by Student's t-test. See Supplementary Table 2 for details on *n* sizes.

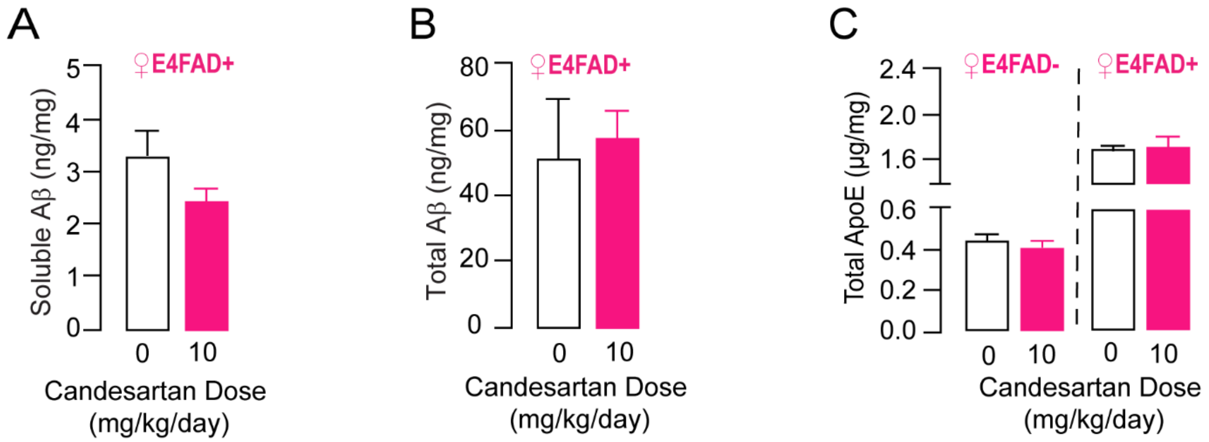

Supplementary Figure 4. Aβ and apoE levels are not modulated by high-dose candesartan treatment in female E4FAD mice. In female E4FAD+ mice treated with 10mg/kg/day candesartan (high dose) there were no differences in **(A)** soluble Aβ42 levels ($t(13)=1.77$, $p>0.10$) when assessed by ELISA analysis compared to vehicle (0mg/kg/day), **(B)** total Aβ42 levels ($t(9)=0.33$, $p=0.75$) compared to vehicle (0mg/kg/day), and **(C)** total apoE levels (E4FAD-: $t(18)=0.76$, $p=46$. E4FAD+: $t(20)=0.13$, $p=89$) compared to vehicle when assessed by ELISA analysis. All data expressed as mean +/- SEM. $p>0.05$ by Student's t-test. See Supplementary Table 2 for details on *n* sizes.

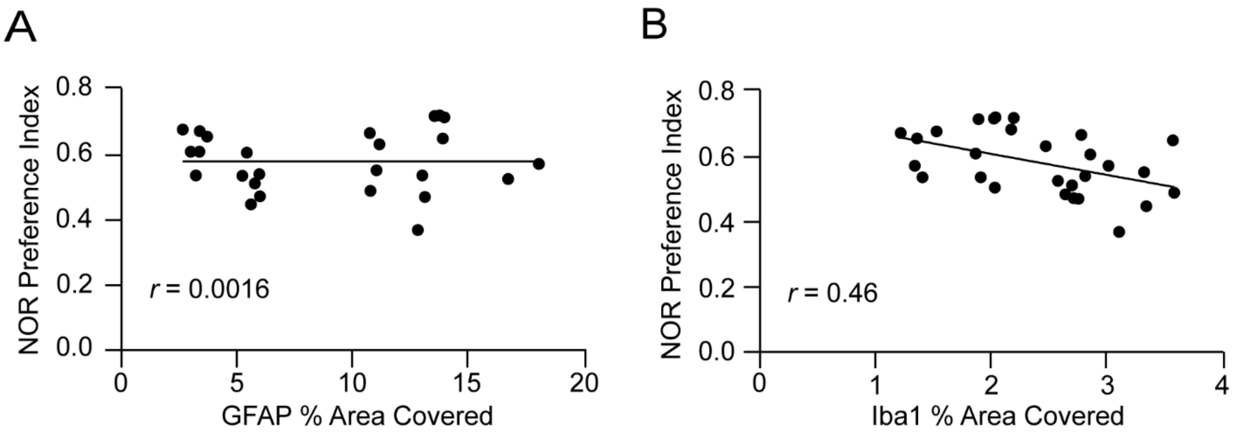

Supplementary Figure 5. Hippocampal levels of Iba-1, but not GFAP, correlate with memory-type behavior in female E4FAD mice. In female E4FAD- and E4FAD+ mice (data combined), **(A)** there was no correlation between hippocampal area immunostained with GFAP and novel object recognition (NOR) preference index scores (r= -0.0016, p=0.99), however **(B)** there was a moderate negative correlation between hippocampal area immunostained with Iba-1 and NOR preference index scores (r= -0.46, p=0.013). Each data point represents a single mouse in the study; p < or > 0.05 by Pearson correlation analysis. See Supplementary Table 2 for details on n sizes.

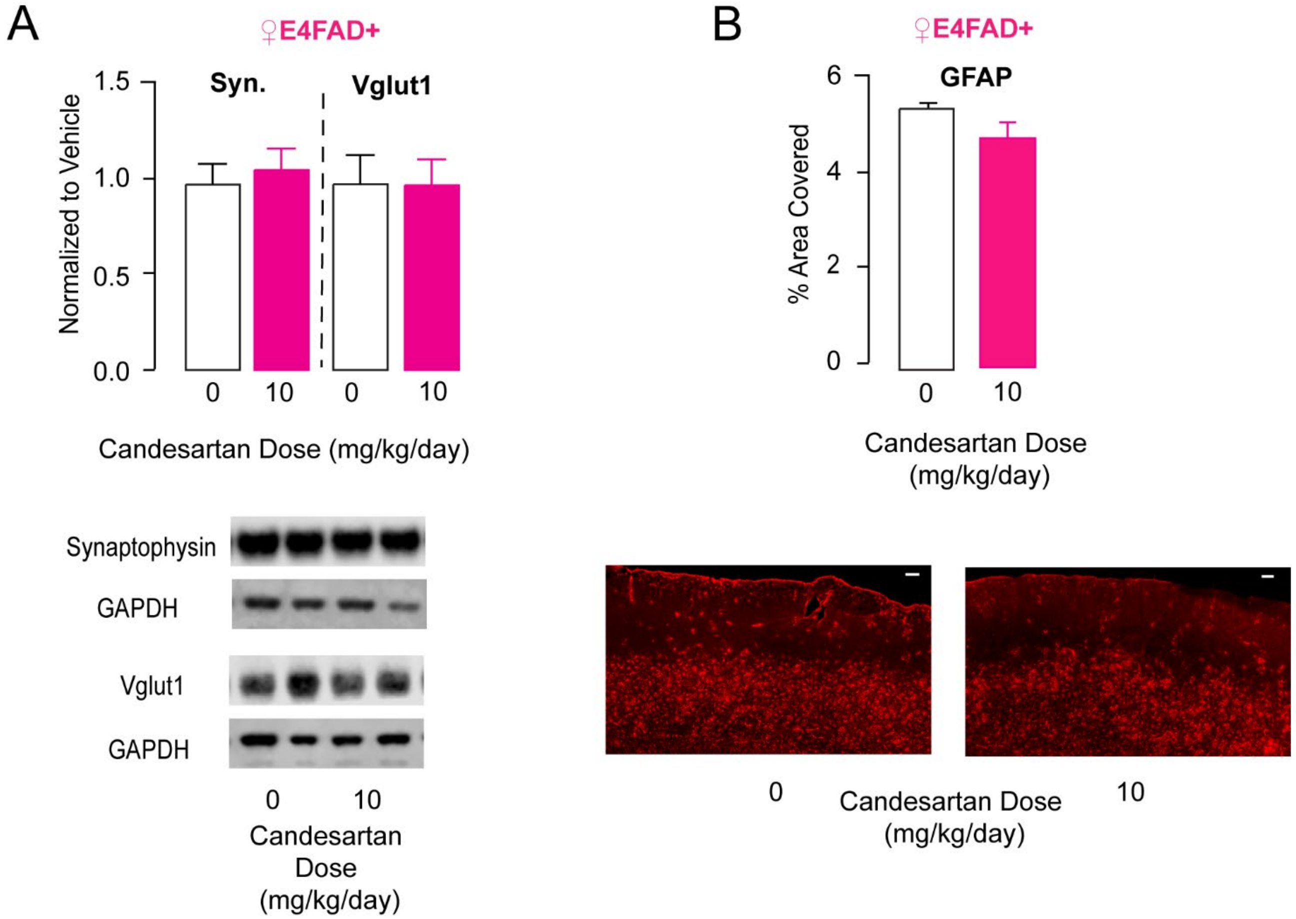

Supplementary Figure 6. High-dose candesartan treatment did not modulate cortical synaptic protein levels or cortical GFAP expression levels compared to vehicle treatment in female E4FAD+ mice. (A) There were no differences in cortical levels of synaptophysin ($t(13)=0.47$, $p=0.64$) or Vglut1 ($t(18)=0.028$, $p=0.97$) between 10mg/kg/day (high dose) candesartan-treated and vehicle-treated female E4FAD+ mice. Quantification of each protein was normalized to GAPDH as a loading control and all data are expressed as a ratio to vehicle-treated mice. **(B)** In female E4FAD+ mice treated with high dose candesartan there were no differences in cortical area immunostained with GFAP compared to vehicle ($t(19)=1.72$, $p=0.10$). Scale bars = 100µm. All data expressed as mean +/- SEM. $p>0.05$ by Student's t-test. See Supplementary Table 2 for details on *n* sizes.

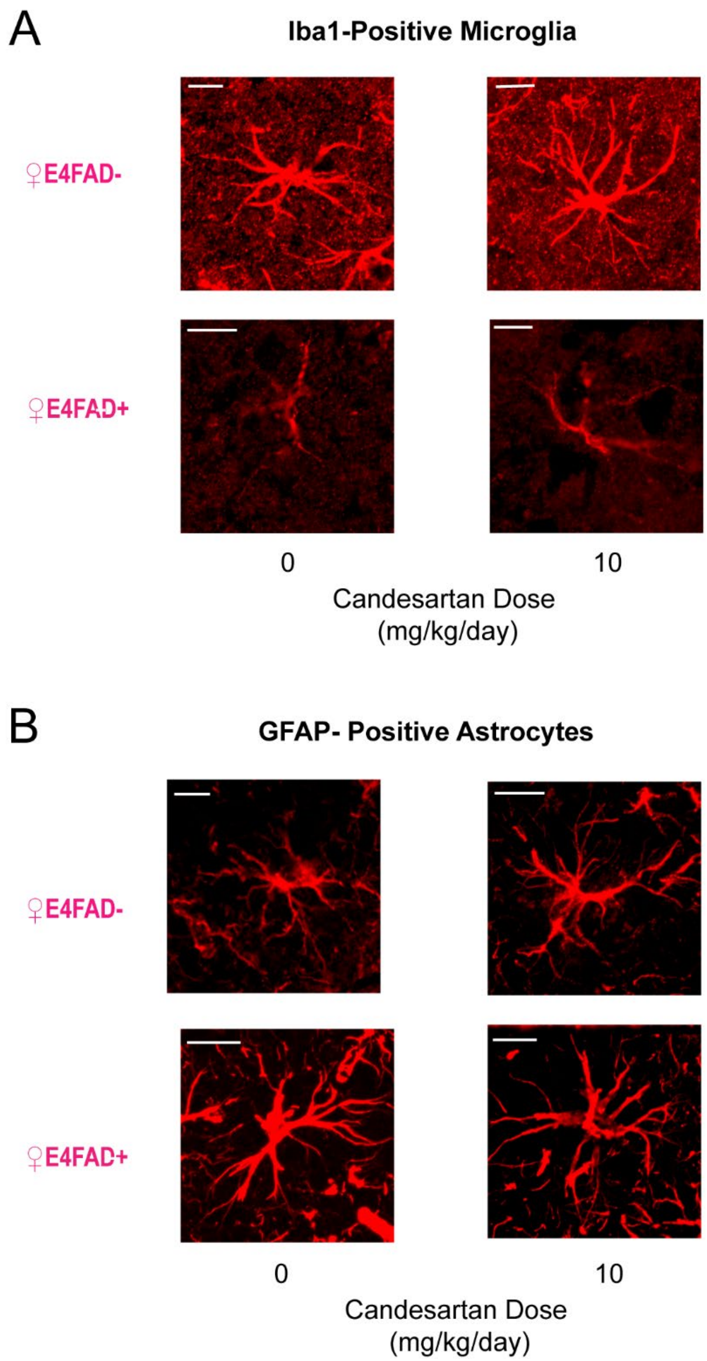

Supplementary Figure 7. High-dose candesartan treatment had no effect on glial morphology in female E4FAD- and E4FAD+ mice. (A) Iba1-positive microglia from female E4FAD- and E4FAD+ mice treated with 10mg/kg/day candesartan did not appear morphologically distinct compared to Iba1-positive microglia from vehicle-treated mice. **(B)** Likewise, GFAP-positive astrocytes from female E4FAD- and E4FAD+ mice treated with 10mg/kg/day candesartan did not appear morphologically distinct compared to GFAP-positive astrocytes from vehicle-treated mice. Scale bars = 10μm.

2 Supplementary Tables

Supplementary Table 1. List of Antibodies Used for Western Blot and IHC Analysis

Target	Vendor	Cat#	Dilution	Application
Rb -α-SV2A (75kDa)	Abcam	Ab32942	1:1000	Western Blot
Rb α -Synaptophysin (38kDa)	Cell Signaling	5461	1:1000	Western Blot
Rb α -SNAP25 (25kDa)	Abcam	Ab5666	1:3000	Western Blot
Rb α -Vglut1 (62kDa)	Abcam	Ab227805	1:1000	Western Blot
Ms α -GAD67 (67kDa)	Abcam	Ab26116	1:1000	Western Blot
Rb α -AGTR1 (50kDa)	Protein Tech	25343	1:1000	Western Blot
Rb α -Angiotensinogen (50kDa)	Abcam	Ab213705	1:1000	Western Blot
Rb α -Angiotensin II Type 2 Receptor (42kDa)	Abcam	Ab92445	1:1000	Western Blot
Ms α -GAPDH (37kDa)	Sigma	G8795	1:4000	Western Blot
800CW Donkey α -Rabbit IgG	LI-COR	926-32213	1:10,000	Western Blot
680RD Goat α -Mouse IgG	LI-COR	926-68070	1:10,000	Western Blot
Gt α -GFAP	Abcam	Ab53554	1:1000	IHC
Rb α -Iba1	Wako	019-19741	1:1000	IHC
Ms α -MOAB2	In House		1:250	IHC
Rb α -Fibrinogen	Agilent Dako	A0080	1:200	IHC
Rt α -CD31	BD Bioscience	550274	1:10	IHC
Dk α -Ms Alexafluor 488	Thermoscientific	A-21202	1:200	IHC
Dk α -Gt Alexafluor 594	Thermoscientific	A11058	1:200	IHC
Gt α -Rb Alexafluor 594	Thermoscientific	A11012	1:200	IHC
Dk α -Rt Alexafluor 488	Thermoscientific	A21208	1:200	IHC

Supplementary Table 2. Statistical Analyses

Readout	Experimental Group	Statistics	Figure Number
Plasma Candesartan Concentration (ng/ml)	Female E4FAD-	***n* sizes:**<br>0mg/kg/day= 3<br>1mg/kg/day= 3<br>10mg/kg/day= 3<br>**One-Way ANOVA**: $F(2, 6)=13.38$, $p=0.0061$<br>**Dunnet's Multiple Comparison's**: Vehicle Control vs. 10mg/kg/d = $p=0.0075$<br>Vehicle Control vs. 1mg/kg/d = $p>0.99$, n.s	Figure 1A
Plasma Candesartan Concentration (ng/ml)	Female E4FAD+	***n* sizes:**<br>0mg/kg/day= 3<br>1mg/kg/day= 3<br>10mg/kg/day= 2<br>**One-Way ANOVA**: $F(2, 5)=19.10$, $p=0.0046$<br>**Dunnet's Multiple Comparison's**: Vehicle Control vs. 10mg/kg/d = $p=0.003$<br>Vehicle Control vs. 1mg/kg/d = $p=0.14$, n.s	Figure 1A
Plasma Candesartan Concentration (ng/ml)	Male E4FAD-	***n* sizes:**<br>0mg/kg/day= 2<br>1mg/kg/day= 3<br>10mg/kg/day= 2<br>**One-Way ANOVA:** $F(2, 4)=324.6$, $p<0.0001$<br>**Dunnet's Multiple Comparison's**: Vehicle Control vs. 10mg/kg/d = $p<0.0001$<br>Vehicle Control vs. 1mg/kg/d = $p=0.96$, n.s	Figure 1A
Plasma Candesartan Concentration (ng/ml)	Male E4FAD+	***n* sizes:**<br>0mg/kg/day= 3<br>1mg/kg/day= 3<br>10mg/kg/day= 3<br>**One-Way ANOVA:** $F(2, 6)= 13.53$, $p=0.006$<br>**Dunnet's Multiple Comparison's**: Vehicle Control vs. 10mg/kg/d = $p=0.0045$<br>Vehicle Control vs. 1mg/kg/d = $p=0.43$, n.s	Figure 1A
Mean Arterial Pressure (mmHg)	Female E4FAD-	***n* sizes:**<br>0mg/kg/day= 6<br>1mg/kg/day= 11<br>10mg/kg/day= 5<br>**One-Way ANOVA**: $F(2, 19)=6.54$, $p=0.0069$<br>**Dunnet's Multiple Comparison's**: Vehicle Control vs. 10mg/kg/d = $p=0.047$<br>Vehicle Control vs. 1mg/kg/d = $p=0.53$, n.s	Figure 1B
Mean Arterial Pressure (mmHg)	Female E4FAD+	***n* sizes:**<br>0mg/kg/day= 5<br>1mg/kg/day= 14<br>10mg/kg/day= 6	Figure 1B

		**One-Way ANOVA**: $F(2, 22)=11.95$, $p=0.0003$<br>**Dunnet's Multiple Comparison's**: Vehicle Control vs. 10mg/kg/d $p=0.0005$<br>Vehicle Control vs. 1mg/kg/d $p=0.42$, n.s	
Mean Arterial Pressure (mmHg)	Male E4FAD-	***n* sizes:**<br>0mg/kg/day= 8<br>1mg/kg/day= 9<br>10mg/kg/day=6<br>**One-Way ANOVA**: $F(2, 20)=14.04$, $p=0.0002$<br>**Dunnet's Multiple Comparison's**: Vehicle Control vs. 10mg/kg/d $p=0.0008$<br>Vehicle Control vs. 1mg/kg/d = $p=0.64$, n.s	Figure 1B
Mean Arterial Pressure (mmHg)	Male E4FAD+	***n* sizes:**<br>0mg/kg/day= 11<br>1mg/kg/day= 11<br>10mg/kg/day= 5<br>**One-Way ANOVA**: $F(2, 24)=4.56$, $p=0.021$<br>**Dunnet's Multiple Comparison's**: Vehicle Control vs. 10mg/kg/d $p=0.011$<br>Vehicle Control vs. 1mg/kg/d = $p=0.37$, n.s	Figure 1B
Novel Object Recognition (Preference Index)	Female E4FAD-	***n* sizes:**<br>0mg/kg/day= 14<br>1mg/kg/day= 4<br>10mg/kg/day= 10<br>**One-Way ANOVA**: $F(2, 25)=15.29$, $p<0.0001$<br>**Dunnet's Multiple Comparison's**: Vehicle Control vs. 10mg/kg/d = $p<0.0001$<br>Vehicle Control vs. 1mg/kg/d = $p=0.47$, n.s	Figure 2A
Novel Object Recognition (Preference Index)	Female E4FAD+	***n* sizes:**<br>0mg/kg/day= 15<br>1mg/kg/day= 16<br>10mg/kg/day= 14<br>**One-Way ANOVA**: $F(2, 42)=14.64$, $p<0.0001$<br>**Dunnet's Multiple Comparison's**: Vehicle Control vs. 10mg/kg/d = $p<0.0001$<br>Vehicle Control vs. 1mg/kg/d = $p=0.55$, n.s	Figure 2A
Novel Object Recognition (Preference Index)	Male E4FAD-	***n* sizes:**<br>0mg/kg/day= 8<br>1mg/kg/day= 8<br>10mg/kg/day= 10<br>**One-Way ANOVA**: $F(2, 23)=1.51$, $p=0.24$, n.s	Figure 2A
Novel Object Recognition (Preference Index)	Male E4FAD+	***n* sizes:**<br>0mg/kg/day= 10<br>1mg/kg/day= 11<br>10mg/kg/day= 8	Figure 2A

		**One-Way ANOVA**: $F(2, 26)=1.23$, $p=0.31$, n.s	
Synaptophysin Western Blot	Female E4FAD-	***n* sizes:**<br>0mg/kg/day= 10<br>10mg/kg/day= 9<br>**Unpaired t test**: $t(17)=2.65$, $p=0.017$	Figure 2B
Synaptophysin Western Blot	Female E4FAD+	***n* sizes:**<br>0mg/kg/day= 10<br>10mg/kg/day= 11<br>**Unpaired t test**: $t(19)=2.89$, $p=0.0092$	Figure 2B
Synaptophysin Western Blot	Male E4FAD-	***n* sizes:**<br>0mg/kg/day= 6<br>10mg/kg/day= 6<br>**Unpaired t test**: $t(10)=0.33$, $p=0.75$, n.s	Figure 2B
Synaptophysin Western Blot	Male E4FAD+	***n* sizes:**<br>0mg/kg/day= 6<br>10mg/kg/day= 5<br>**Unpaired t test**: $t(9)=0.34$, $p=0.74$, n.s	Figure 2B
SV2A Western Blot	Female E4FAD-	***n* sizes:**<br>0mg/kg/day= 9<br>10mg/kg/day= 8<br>**Unpaired t test**: $t(15)=2.33$, $p=0.034$	Figure 2B
SV2A Western Blot	Female E4FAD+	***n* sizes:**<br>0mg/kg/day= 9<br>10mg/kg/day= 10<br>**Unpaired t test**: $t(17)=2.83$, $p=0.012$	Figure 2B
SV2A Western Blot	Male E4FAD-	***n* sizes:**<br>0mg/kg/day= 6<br>10mg/kg/day= 6<br>**Unpaired t test**: $t(10)=0.43$, $p=0.67$, n.s	Figure 2B
SV2A Western Blot	Male E4FAD+	***n* sizes:**<br>0mg/kg/day= 6<br>10mg/kg/day= 5<br>**Unpaired t test**: $t(9)=0.66$, $p=0.53$, n.s	Figure 2B
SNAP25 Western Blot	Female E4FAD-	***n* sizes:**<br>0mg/kg/day= 10<br>10mg/kg/day= 9<br>**Unpaired t test**: $t(17)=1.45$, $p=0.16$, n.s	Figure 2B
SNAP25 Western Blot	Female E4FAD+	***n* sizes:**<br>0mg/kg/day= 10<br>10mg/kg/day= 12<br>**Unpaired t test**: $t(20)=3.03$, $p=0.0067$	Figure 2B
SNAP25 Western Blot	Male E4FAD-	***n* sizes:**<br>0mg/kg/day= 6<br>10mg/kg/day= 6<br>**Unpaired t test**: $t(10)=0.25$, $p=0.81$, n.s	Figure 2B
SNAP25 Western Blot	Male E4FAD+	***n* sizes:**<br>0mg/kg/day= 6	Figure 2B

		10mg/kg/day= 5<br>**Unpaired t test**: *t*(9)=0.56, *p*=0.56, n.s	
Angiotensin II ELISA	Female E4FAD-	***n* sizes:**<br>0mg/kg/day= 10<br>10mg/kg/day= 10<br>**Unpaired t test**: *t*(18)=0.15, *p*=0.88, n.s	Figure 3A
Angiotensin II ELISA	Female E4FAD+	***n* sizes:**<br>0mg/kg/day= 10<br>10mg/kg/day= 9<br>**Unpaired t test**: *t*(17)=1.85, *p*=0.081, n.s	Figure 3A
Angiotensin II ELISA	Male E4FAD-	***n* sizes:**<br>0mg/kg/day= 5<br>10mg/kg/day= 5<br>**Unpaired t test**: *t*(8)=2.23, *p*=0.056, n.s	Figure 3A
Angiotensin II ELISA	Male E4FAD+	***n* sizes:**<br>0mg/kg/day= 5<br>10mg/kg/day= 5<br>**Unpaired t test**: *t*(8)=1.53, *p*=0.16, n.s	Figure 3A
Angiotensinogen Western Blot	Female E4FAD-	***n* sizes:**<br>0mg/kg/day= 10<br>10mg/kg/day= 9<br>**Unpaired t test**: *t*(17)=1.30, *p*=0.21, n.s	Figure 3B
Angiotensinogen Western Blot	Female E4FAD+	***n* sizes:**<br>0mg/kg/day= 12<br>10mg/kg/day= 12<br>**Unpaired t test**: *t*(22)=0.81, *p*=0.43, n.s	Figure 3B
Angiotensinogen Western Blot	Male E4FAD-	***n* sizes:**<br>0mg/kg/day= 6<br>10mg/kg/day= 6<br>**Unpaired t test**: *t*(10)=0.27, *p*=0.79, n.s	Figure 3B
Angiotensinogen Western Blot	Male E4FAD+	***n* sizes:**<br>0mg/kg/day= 6<br>10mg/kg/day= 5<br>**Unpaired t test**: *t*(9)=1.19, *p*=0.26, n.s	Figure 3B
AT1R Western Blot	Female E4FAD-	***n* sizes:**<br>0mg/kg/day= 10<br>10mg/kg/day= 10<br>**Unpaired t test**: *t*(18)=1.00, *p*=0.33, n.s	Figure 3B
AT1R Western Blot	Female E4FAD+	***n* sizes:**<br>0mg/kg/day= 12<br>10mg/kg/day= 11<br>**Unpaired t test**: *t*(21)=0.09, *p*=0.92, n.s	Figure 3B
AT1R Western Blot	Male E4FAD-	***n* sizes:**<br>0mg/kg/day= 6<br>10mg/kg/day= 5<br>**Unpaired t test**: *t*(10)=2.28, *p*=0.073, n.s	Figure 3B
AT1R Western Blot	Male E4FAD+	***n* sizes:**	Figure 3B

		0mg/kg/day= 6<br>10mg/kg/day= 5<br>**Unpaired t test**: $t(9)=1.44$, $p=0.18$, n.s	
AT2R Western Blot	Female E4FAD-	***n* sizes:**<br>0mg/kg/day= 10<br>10mg/kg/day= 10<br>**Unpaired t test**: $t(18)=1.00$, $p=0.33$, n.s	Figure 3B
AT2R Western Blot	Female E4FAD+	***n* sizes:**<br>0mg/kg/day= 12<br>10mg/kg/day= 12<br>**Unpaired t test**: $t(22)=0.21$, $p=0.84$, n.s	Figure 3B
AT2R Western Blot	Male E4FAD-	***n* sizes:**<br>0mg/kg/day= 6<br>10mg/kg/day= 6<br>**Unpaired t test**: $t(10)=1.04$, $p=0.32$, n.s	Figure 3B
AT2R Western Blot	Male E4FAD+	***n* sizes:**<br>0mg/kg/day= 6<br>10mg/kg/day= 5<br>**Unpaired t test**: $t(9)=1.45$, $p=0.17$, n.s	Figure 3B
IHC stain for Iba-1	Female E4FAD-	***n* sizes:**<br>0mg/kg/day= 9<br>10mg/kg/day= 9<br>**Unpaired t test**: $t(16)=6.76$, $p<0.0001$	Figure 4A
IHC stain for Iba-1	Female E4FAD+	***n* sizes:**<br>0mg/kg/day= 10<br>10mg/kg/day= 10<br>**Unpaired t test**: $t(18)=5.08$, $p<0.0001$	Figure 4A
IHC stain for GFAP	Female E4FAD-	***n* sizes:**<br>0mg/kg/day= 9<br>10mg/kg/day= 9<br>**Unpaired t test**: $t(16)=15.06$, $p<0.0001$	Figure 4B
IHC stain for GFAP	Female E4FAD+	***n* sizes:**<br>0mg/kg/day= 10<br>10mg/kg/day= 10<br>**Unpaired t test**: $t(18)=2.44$, $p=0.025$	Figure 4B
IHC stain for CD31	Female E4FAD-	***n* sizes:**<br>0mg/kg/day= 9<br>10mg/kg/day= 9<br>**Unpaired t test**: $t(16)=0.28$, $p=0.78$, n.s	Figure 5A
IHC stain for CD31	Female E4FAD+	***n* sizes:**<br>0mg/kg/day= 10<br>10mg/kg/day= 10<br>**Unpaired t test**: $t(18)=0.003$, $p=0.99$, n.s	Figure 5A
IHC stain for Fibrinogen	Female E4FAD-	***n* sizes:**<br>0mg/kg/day= 8<br>10mg/kg/day= 8<br>**Unpaired t test**: $t(15)=0.47$, $p=0.64$, n.s	Figure 5B

IHC stain for Fibrinogen	Female E4FAD+	***n* sizes:**<br>0mg/kg/day= 9<br>10mg/kg/day= 9<br>**Unpaired t test**: $t(14)=0.23$, $p=0.82$, n.s	Figure 5B
IHC stain for MOAB-2	Female E4FAD+	***n* sizes:**<br>0mg/kg/day= 10<br>10mg/kg/day= 10<br>**Unpaired t test**: $t(18)=0.74$, $p=0.47$, n.s	Figure 5C
Open Field (Total Distance)	Female E4FAD-	***n* sizes:**<br>0mg/kg/day= 12<br>1mg/kg/day= 2<br>10mg/kg/day= 10<br>**One-Way ANOVA:** $F(2,21)=0.11$, $p=0.89$, n.s	Supplementary Figure 2A
Open Field (Total Distance)	Female E4FAD+	***n* sizes:**<br>0mg/kg/day= 10<br>1mg/kg/day= 14<br>10mg/kg/day= 16<br>**One-Way ANOVA**: $F(2, 37)=7.19$, $p=0.0023$<br>**Dunnet's Multiple Comparison's**: Vehicle Control vs. 10mg/kg/d = $p>0.05$, n.s<br>Vehicle Control vs. 1mg/kg/d = $p=0.0014$	Supplementary Figure 2A
Open Field (Total Distance)	Male E4FAD-	***n* sizes:**<br>0mg/kg/day= 12<br>1mg/kg/day= 8<br>10mg/kg/day= 13<br>**One-Way ANOVA:** $F(2,30)=1.19$, $p=0.32$, n.s	Supplementary Figure 2A
Open Field (Total Distance)	Male E4FAD+	***n* sizes:**<br>0mg/kg/day= 9<br>1mg/kg/day= 12<br>10mg/kg/day= 8<br>**One-Way ANOVA:** $F(2,26)=0.16$, $p=0.85$, n.s	Supplementary Figure 2A
Open Field (% Distance in Perimeter)	Female E4FAD-	***n* sizes:**<br>0mg/kg/day= 12<br>1mg/kg/day= 2<br>10mg/kg/day= 10<br>**One-Way ANOVA:** $F(2,22)=0.05$, $p=0.95$, n.s	Supplementary Figure 2B
Open Field (% Distance in Perimeter)	Female E4FAD+	***n* sizes:**<br>0mg/kg/day= 10<br>1mg/kg/day= 14<br>10mg/kg/day= 16<br>**One-Way ANOVA:** $F(2,36)=1.71$, $p=0.19$, n.s	Supplementary Figure 2B
Open Field (% Distance in Perimeter)	Male E4FAD-	***n* sizes:**<br>0mg/kg/day= 12<br>1mg/kg/day= 8<br>10mg/kg/day= 13<br>**One-Way ANOVA:** $F(2,30)=0.50$, $p=0.61$, n.s	Supplementary Figure 2B

Open Field (% Distance in Perimeter)	Male E4FAD+	***n* sizes:**<br>0mg/kg/day= 9<br>1mg/kg/day= 12<br>10mg/kg/day= 8<br>**One-Way ANOVA:** *F*(2,26)=3.69, *p*=0.039<br>**Dunnet's Multiple Comparison's**: Vehicle Control vs. 10mg/kg/d = *p*>0.05, n.s<br>Vehicle Control vs. 1mg/kg/d = *p*>0.05, n.s	Supplementary Figure 2B
Vglut1 Western Blot	Female E4FAD-	***n* sizes:**<br>0mg/kg/day= 10<br>10mg/kg/day= 9<br>**Unpaired t test**: *t*(17)=0.69, *p*=49, n.s	Supplementary Figure 3
Vglut1 Western Blot	Female E4FAD+	***n* sizes:**<br>0mg/kg/day= 11<br>10mg/kg/day= 12<br>**Unpaired t test**: *t*(21)=1.87, *p*=0.075, n.s	Supplementary Figure 3
Vglut1 Western Blot	Male E4FAD-	***n* sizes:**<br>0mg/kg/day= 6<br>10mg/kg/day= 6<br>**Unpaired t test**: *t*(10)=0.54, *p*=0.60, n.s	Supplementary Figure 3
Vglut1 Western Blot	Male E4FAD+	***n* sizes:**<br>0mg/kg/day= 6<br>10mg/kg/day= 5<br>**Unpaired t test**: *t*(9)=0.76, *p*=0.46, n.s	Supplementary Figure 3
GAD67 Western Blot	Female E4FAD-	***n* sizes:**<br>0mg/kg/day= 10<br>10mg/kg/day= 9<br>**Unpaired t test**: *t*(17)=0.73, *p*=0.48, n.s	Supplementary Figure 3
GAD67 Western Blot	Female E4FAD+	***n* sizes:**<br>0mg/kg/day= 9<br>10mg/kg/day= 10<br>**Unpaired t test**: *t*(17)=0.66, *p*=0.52, n.s	Supplementary Figure 3
GAD67 Western Blot	Male E4FAD-	***n* sizes:**<br>0mg/kg/day= 6<br>10mg/kg/day= 6<br>**Unpaired t test**: *t*(10)=0.22, *p*=0.83, n.s	Supplementary Figure 3
GAD67 Western Blot	Male E4FAD+	***n* sizes:**<br>0mg/kg/day= 6<br>10mg/kg/day= 5<br>**Unpaired t-test**: *t*(9)=0.95, *p*=0.36, n.s	Supplementary Figure 3
Aβ42 ELISA (Soluble)	Female E4FAD+	***n* sizes:**<br>0mg/kg/day= 6<br>10mg/kg/day= 9<br>**Unpaired t-test**: *t*(13)=1.77, *p*=0.10, n.s	Supplementary Figure 4A
Aβ42 ELISA (Total)	Female E4FAD+	***n* sizes:**<br>0mg/kg/day= 5<br>10mg/kg/day= 6<br>**Unpaired t-test**: *t*(9)=0.33, *p*=0.75, n.s	Supplementary Figure 4B

ApoE ELISA	Female E4FAD-	***n* sizes:**<br>0mg/kg/day= 10<br>10mg/kg/day= 10<br>**Unpaired t-test**: $t(18)=0.76$, $p=0.46$, n.s	Supplementary Figure 4C
ApoE ELISA	Female E4FAD+	***n* sizes:**<br>0mg/kg/day= 11<br>10mg/kg/day= 11<br>**Unpaired t-test**: $t(20)=0.13$, $p=0.89$, n.s	Supplementary Figure 4C
Novel Object Recognition (Preference Index) and GFAP Correlation	Female E4FAD-<br>Female E4FAD+	***n* sizes:**<br>0mg/kg/day (E4FAD-) = 6<br>0mg/kg/day (E4FAD+) = 7<br>10mg/kg/day (E4FAD-) = 6<br>10mg/kg/day (E4FAD+) = 6<br>**Pearson Correlation:** $r= -0.0016$, $p=0.99$, n.s	Supplementary Figure 5A
Novel Object Recognition (Preference Index) and Iba1 Correlation	Female E4FAD-<br>Female E4FAD+	***n* sizes:**<br>0mg/kg/day (E4FAD-) = 7<br>0mg/kg/day (E4FAD+) = 7<br>10mg/kg/day (E4FAD-) = 6<br>10mg/kg/day (E4FAD+) = 8<br>**Pearson Correlation:** $r= -0.46$, p=0.0.013	Supplementary Figure 5B
Synaptophysin Western Blot (cortex)	Female E4FAD+	***n* sizes:**<br>0mg/kg/day = 8<br>10mg/kg/day = 7<br>**Unpaired t-test:** $t(13)=0.47$, $p=0.64$, n.s	Supplementary Figure 6A
Vglut1 Western Blot (cortex)	Female E4FAD+	***n* sizes:**<br>0mg/kg/day = 9<br>10mg/kg/day = 11<br>**Unpaired t-test:** $t(18)=0.028$, $p=0.97$, n.s	Supplementary Figure 6A
IHC stain for GFAP (cortex)	Female E4FAD+	***n* sizes:**<br>0mg/kg/day = 10<br>10mg/kg/day = 10<br>**Unpaired t-test:** $t(19)=1.72$, $p=0.10$, n.s	Supplementary Figure 6B

Supplementary Table 3. Hippocampal cytokine/chemokine levels in female E4FAD- and E4FAD+ mice

Cytokine/Chemokine Mean (SEM)	Female E4FAD+ 0mg/kg/day Candesartan	Female E4FAD+ 10mg/kg/day Candesartan	Female E4FAD- 0mg/kg/day Candesartan	Female E4FAD- 10mg/kg/day Candesartan
GCSF	81.44 (5.06)	64.09* (4.55)	54.52 (5.71)	60.40 (5.20)
Eotaxin	1.21 (0.06)	1.11 (0.07)	1.03 (0.08)	1.08 (0.07)
GMCSF	14.08 (0.56)	12.15 (0.84)	11.35 (1.10)	12.26 (0.72)
IL1α	33.96 (1.29)	33.38 (1.49)	31.32 (2.38)	33.38 (2.03)
IL1β	1.54 (0.08)	1.59 (0.12)	1.95 (0.45)	2.90 (0.80)
IL2	9.67 (0.36)	9.71 (0.41)	10.45 (1.61)	12.18 (1.81)
IL7	1.75 (0.08)	1.68 (0.09)	1.74 (0.12)	1.72 (0.08)
IL9	2731.47 (120.61)	3214.75 (371.17)	3424.97 (1239)	4724.20 (1244.75)
IL10	17.54 (0.94)	17.22 (0.82)	16.01 (1.21)	16.93 (0.71)
IL12p40	7.01 (0.38)	7.03 (0.43)	6.11 (0.57)	6.42 (0.52)
IL12p70	9.06 (0.35)	7.55 (0.63)	6.66 (0.63)	7.12 (0.65)
IL13	296.58 (29.71)	298.18 (37.05)	356.99 (104.67)	540.83 (160.37)
IL17	4.32 (0.17)	3.90 (0.21)	3.73 (0.25)	3.75 (0.19)
IP10	19.96 (1.26)	20.15 (3.89)	5.97 (0.41)	6.83 (0.49)
KC	6.14 (0.31)	6.13 (0.35)	3.95 (0.85)	5.83 (1.74)
MIP1α	23.14 (0.93)	24.13 (1.54)	28.30 (7.27)	40.00 (10.91)
MCSF	3.98 (0.16)	3.82 (0.14)	1.38 (0.09)	1.50 (0.13)
MIP2	15.35 (0.68)	14.12 (0.50)	12.51 (1.12)	13.60 (1.31)
MIG	25.93 (4.43)	20.96 (4.00)	5.03 (0.53)	5.43 (0.68)
VEGF	27.79 (1.94)	29.24 (1.11)	16.16 (1.82)	17.90 (1.80)

RANTES	3.60 (0.17)	3.39 (0.13)	3.07 (0.32)	2.86 (0.15)
IFNγ	12.80 (0.68)	11.55 (1.18)	8.80 (1.38)	10.76 (0.67)
IL6	20.77 (1.07)	20.05 (1.12)	12.45 (2.00)	14.45 (1.23)
IL15	12.03 (0.73)	10.36 (0.82)	8.54 (0.81)	9.40 (0.84)
